# Concordance in a World without a Gold Standard: A New Non-Invasive Methodology for Improving Accuracy of Fibrosis Markers

**DOI:** 10.1371/journal.pone.0003857

**Published:** 2008-12-04

**Authors:** Thierry Poynard, Patrick Ingiliz, Laure Elkrief, Mona Munteanu, Pascal Lebray, Rachel Morra, Djamila Messous, Francoise Imbert Bismut, Dominique Roulot, Yves Benhamou, Dominique Thabut, Vlad Ratziu

**Affiliations:** 1 APHP Groupe Hospitalier Pitié-Salpêtrière, Université Paris VI, CNRS UMR, Paris, France; 2 Biopredictive, Paris, France; 3 APHP Hopital Avicennes, Bobigny, France; University of Michigan, United States of America

## Abstract

**Background:**

Assessing liver fibrosis is traditionally performed by biopsy, an imperfect gold standard. Non-invasive techniques, liver stiffness measurements (LSM) and biomarkers [FibroTest® (FT)], are widely used in countries where they are available. **The aim** was to identify factors associated with LSM accuracy using FT as a non-invasive endpoint and vice versa.

**Methods:**

The proof of concept was taken using the manufacturers recommendations for excluding patients at high risk of false negative/positive. The hypothesis was that the concordance between LSM and FT, would be improved by excluding high-risk patients. Thereafter, the impact of potential variability factors was assessed by the same methods. Liver biopsy and independent endpoints were used to validate the results.

**Results:**

Applying manufacturers' recommendations in 2,004 patients increased the strength of concordance between LSM and FT (P<0.00001). Among the 1,338 patients satisfying recommendations, the methodology identified a significant LSM operator effect (P = 0.001) and the following variability factors (all P<0.01), related to LSM: male gender, older age, and NAFLD as a cause of liver disease. Biopsy confirmed in 391 patients these results.

**Conclusion:**

This study has validated the concept of using the strength of concordance between non-invasive estimates of liver fibrosis for the identification of factors associated with variability and precautions of use.

## Introduction

A major clinical challenge is finding the best means of evaluating and managing the increasing numbers of patients with chronic liver disease [1]. Liver biopsy, due to its risks and limitations, is no longer considered mandatory as the first-line indicator of liver injury, and several markers have been developed as non-invasive alternatives [1], [2].

The true liver disease status, the “gold standard” is the histological analysis of nearly the entire liver [3]. Therefore the definitive diagnosis is impossible to obtain in routine practice, and the liver biopsy, an “imperfect gold standard” [4], is used as a standard against which new tests are evaluated [5], [6].

The assessment of liver fibrosis by non-invasive techniques, biomarkers [FibroTest® (FT)] [7], [8] and liver stiffness measurements (LSM) by Fibroscan® [9], [10] is now widely done in countries where these techniques are available and approved.[11], [12] It is therefore essential to identify factors associated with variability of these imperfect gold standards to reduce the risk of false positive and false negative.

The aim was to propose an original methodology for identifying factors associated with the variability of these techniques.

## Methods

### Concept

We developed the following concept: when there are no perfect gold standards but only imperfect gold standards for estimating the truth, the measurement of the strength of the concordance between these imperfect gold standards could be used as a tool for identifying factors of variability.

Any variability factor of one test should impact the strength of the association between the two tests assuming that this variability factor is not also associated with the other test (independent tests).

The following examples illustrate this concept. For estimating liver fibrosis stages, LSM and FT have been considered as two validated imperfect gold standards. [7]–[12] One variability factor of LSM, [13]–[14] is the total number of valid measurements. Therefore if subjects with a number of measurements below the recommended number (n = 10) are included, the strength of association between LSM and FT fibrosis estimates should decrease in comparison with a population excluding these subjects. The tests are independent as there is no rationale suggesting that the number of valid measurements could be associated with the FT estimate.

Similarly one variability factor of FT, [7]–[8], [15] is the presence of Gilbert's syndrome (genetic increase of total bilirubin) that induces a risk of FT false positive as bilirubin is a component of FT. Therefore if subjects with Gilbert syndrome are included, the strength of association between LSM and FT should decrease in comparison with a population excluding these subjects. The tests are independent as there is no rational suggesting that the presence of Gilbert syndrome could be related to the LSM estimate.

### Proof of concept

To validate this concept we compared the strength of concordance between LSM and FT, for the diagnosis of advanced fibrosis stage, between a population including only subjects who fulfilled the quality criteria as recommended by the manufacturer (low risk profile of false negatives/positives) and a population not fulfilling these quality criteria (high risk of false negatives/positives).

For LSM the recommended criteria were: success rate greater than 60% (SR60), at least 10 valid liver stiffness measurements (V10) and interquartile range/median LSM<30% (IQR30) [12]–[15]; For FT, these were: a security algorithm profile excluding Gilbert's disease, hemolysis, acute inflammation profiles and extremes values (one percentile) of FT components [8], [12], [16].

### Potential factors of variability

The following potential factors of LSM variability were tested: operator effect, male gender, steatosis (presumed with SteatoTest), necroinflammatory activity (presumed with ActiTest), normal transaminases ALT, anthropometric factors (BMI, abdominal and thoracic folds, waist circumference), cause of liver disease and ethnic origin.

The following potential factors of FT variability were tested: normal transaminases ALT, cause of liver disease and ethnic origin.

### Validation of results

Liver biopsy was used to confirm the results observed with the proposed methods.

In order to attribute the cause of “cirrhosis discordance” between FT and LSM, all the cases with cirrhosis predicted by only one method were reviewed as previously published [5].

### Biochemical markers

FibroTest, ActiTest and SteatoTest (Biopredictive, Paris, France) were performed according to published recommendations. [25], [8], [16]–[18]

### Liver stiffness measurements

Patients were studied using the non-invasive method of transient elastography (Fibroscan, Echosens, Paris, France). The stiffness results are expressed in kilopascals (kPa). The technique was performed by trained (more than 100 measurements) senior hepatologists (operator), blinded to all other characteristics, and according to the manufacturers' recommendations. The following recommended cutoffs were used: 5.1, 8.8, 9.5 and 14.5 kPa for the F0, F1, F2, F3 and F4 staging respectively [14]. We took 8.8 as cutoff for advanced fibrosis (defined as F2 or greater) and not 7.1 kPa [9] as the 95% percentiles of healthy population is 7.8 for female and 8.0 for male [19].

### Biopsy

Staging and grading were performed blinded to non-invasive methods, according to METAVIR scoring system [20] and according to Brunt et al for NAFLD [21] by one experienced pathologist.

### Statistical analysis

The strength of concordance between LSM and FT was assessed using six methods, three categorical [the area under the receiver operating characteristic curve (AUC), the kappa reliability test (K) for 2 (K2) fibrosis stages], two quantitative [Spearman coefficient of correlation (R), with partial correlation (pR) when necessary, the intraclass coefficient of correlation (ICC)] and one mixed (the regression curve randomization test) [22]–[27].

Concordance analyses were performed with NCSS software (Kaysville, Utah, USA) [27].

The program “TAGS” was used for the evaluation of FT and LSM accuracy, in the absence of a perfect gold standard [28]. The model used two populations comparing FT and LSM, without the perfect disease status, the present data set (n = 1,109 patients) and the data published by Castera et al (n = 183)[9], and two disease free reference populations (925 blood donors and 429 healthy subjects) [17], [19].

A sensitivity analysis has been performed using the LSM 7.1 cutoff [9] also used for advanced fibrosis, instead of 8.8 kPa [14]. Details of methods are given in supporting information file Text S1.

Because of the number of statistical comparisons and in order to decrease the risk of false positive conclusion, a P value lower than 0.01 using three different concordance statistical methods were needed to conclude at a significant difference.

## Results

The database included 2,004 consecutive patients who underwent simultaneously LSM and FT (Table 1) in our center between June 2005 and April 2007.

**Table 1:** Characteristics of included patients.

Characteristics	
**Number of patients**	**1338**
Age at serum, years, mean (SD)	50 (13)
Male (%)	822 (61%)
*Ethnic origin*	
Caucasian	956 (71.5%)
Asian	100 (7.5%)
North African	140 (10.5%)
Other African	142 (10.5%)
*Anthropometric data*$	
Height m	1.7 (0.1)
Weight kg	70 (14)
BMI, kg/m2	24 (4)
Abdominal fold mm	21 (12)
Thoracic fold mm	12 (7)
Waist circumference cm	86 (12)
*Daily alcohol > = 30 g/day*	*58 (4%)*
***Diagnosis***	
**Chronic disease**	100%
HCV	517 (39%)
HBV	255 (19%)
NAFLD	168 (13%)
HIV coinfection	112 (8%)
ALD	32 (2%)
Other	99 (7%)
Unknown	155 (12%)
***Biochemistry***	
ALT IU/L	71 (204)
AST IU/L	50 (41)
Cholesterol mmol/L	4.7 (1)
Glucose mmol/L	5.3 (1.8)
Triglycerides mmol/L	1.2 (0.8)
FibroTest	0.43 (0.27)
ActiTest	0.35 (0.26)
SteatoTest	0.35 (0.23)

A total of 604 (30%) not fulfilled the recommended criteria for LSM interpretation, 88 (4%) not fulfilled the criteria for FT interpretation and 1338 (67%) fulfilled both criteria. Patients with non-interpretable LSM were older, more often female, and had higher weight, BMI, and abdominal and thoracic folds in comparison with patients with interpretable LSM (Table 1 and supporting Table S1).

### Proof of concept

The manufacturers' recommended criteria were all associated with the strength of concordance between FT and LSM. (Table 2 and supporting Table S2) The proof of concept was validated, as applying the manufacturers' recommendations significantly increased the strength of concordance between LSM and FT, using all statistical methods (from P = 0.04 to P<0.00001).

**Table 2:** Proof of concept: manufacturers' risk factors of false positives/negatives are associated with strength of concordance between FibroTest (FT) and liver stiffness measurements (LSM).

	Method assessing	concordance				
Characteristics (number patients)	AUROC*	Kappa 2	Kappa 3	Spearman	Intra Class Coefficient	Curve fitting
	Advanced versus non advanced fibrosis	Advanced versus non advanced fibrosis	F0F1 vs F2F3 vs F4	FT vs LSM	FT vs LSM	Curve inequality
	Mean (95% CI) Significance	Kappa Mean (se)	Mean (se)	Mean (95% CI)	Mean (95% CI)	Linear-Linear Model F-test R2
**All patients (2004)**	0.72 (0.70–0.75)	0.38 (0.02)	0.29 (0.02)	0.44 (0.41–0.48)	0.46 (0.42–0.49)	0.21
**Manufacturer risk factors ** **	**P<0.0001**	**P = 0.01**	**P = 0.04**	**P = 0.001**	**P = 0.001**	**P<0.00001**
**Yes (666)**	**0.63 (0.59–0.67)**	**0.29 (0.04)**	**0.20 (0.03)**	**0.27 (0.20–0.34)**	**0.19 (0.12–0.26)**	**0.09**
**No (1338)**	**0.78 (0.76–0.81)**	**0.42 (0.03)**	**0.33 (0.02)**	**0.55 (0.51–0.59)**	**0.52 (0.48–0.56)**	**0.34**
**High risk Elastography**	P<0.0001	P = 0.08	P = 0.003	P = 0.001	P<0.0001	P<0.00001
**Yes** (604)	0.63 (0.58–0.68)	0.32 (0.04)	0.21 (0.03)	0.26 (0.19–0.33)	−0.04 (−0.12–0.04)	0.10
**No** (1400)	0.78 (0.75–0.80)	0.40 (0.025)	0.32 (0.02)	0.54 (0.51–0.58)	0.47 (0.43–0.51)	0.33
**High risk FibroTest**	P = 0.09	P = 0.01	P = 0.09	P = 0.40	P = 0.10	P<0.00001
Yes (88)	0.68 (0.55–0.78)	0.15 (0.07)	0.16 (0.06)	0.30 (0.10–0.48)	0.23 (0.02–0.41)	0.11
**No** (1916)	0.78 (0.75–0.80)	0.39 (0.02)	0.29 (0.016)	0.44 (0.41–0.48)	0.46 (0.37–0.45)	0.22

* FT as enpoint for LSM AUROCs, LSM as endpoint for FT AUROCs.

** If LSM was taken as endpoint same results were observed: AUROC of patients with manufacturer risk factors (n = 666) was 0.71 (0.67–0.75) vs 0.81 (0.78–0.84) in patients without risk factors (n = 1338; P = 0.0001).

### Factors associated with strength of concordance

**Figure 1:**
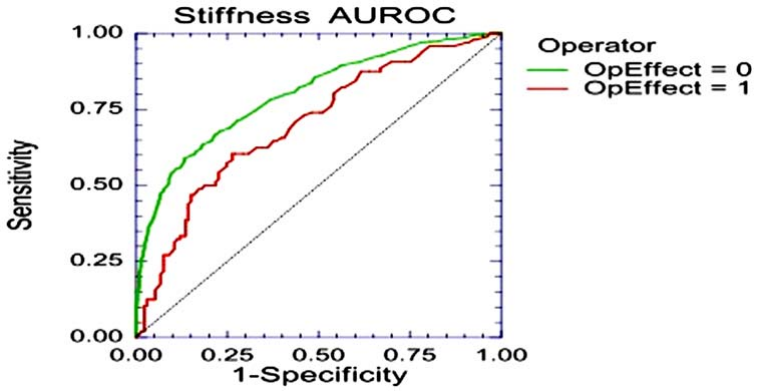
Operator effect. One operator has significantly lower concordance between stiffness measurement and FibroTest than the other operator (area under the ROC curve = 0.70; 95%CI 0.63–0.76, versus 0.80; 95%CI 0.77–0.82; P = 0.009).

A significant operator effect was identified with weaker concordance than all the other operators in the population without high-risk profile (Figure 1). After excluding the 375 patients analyzed by this operator the strength of concordance was significantly higher in the 1,109 remaining patients. In the pre-included population this operator had a higher percentage of LSM high-risk profile 36% (135/375) vs 29% (469/1629; P = 0.006) among the other operators. The major risk factor was IQR/LSM>30% observed in 25% (94/375) vs 18% (287/1629; P = 0.0009) among the other operators. The percentages of other risk factors were not different including the number of valid measurements, success rate, FT risk profile, previous experience of LSM, and prevalence of possible risk factors (data not shown).

Among the 1,109 patients with homogeneous operators and recommended criteria, the following factors were significantly associated with lower strength of concordance using at least three methods and protected for multiple testing: older age (Kappa = 0.37 if 50 years or older versus kappa = 0.50 if younger), NAFLD as a cause of chronic liver disease (Kappa = 0.24 for NAFLD versus kappa = 0.40 for other disease), the absence of steatosis presumed with SteatoTest (Kappa = 0.38 in the absence of steatosis and kappa = 0.59 in the presence of steatosis; inverse than the prior hypothesis).

The following factors were associated with lower strength of concordance (only for one or two methods or P value greater than 0.01): male gender, BMI greater than 30 kg/m2, higher weight, abdominal fold >30 mm, thoracic fold >15 mm, higher waist circumference in male, African ethnic origin, and non-elevated ALT values. There was no impact on the strength of concordance for height, and daily alcohol consumption (supporting Table S3).

### Validation using liver biopsy

A total of 391 patients had previously undergone liver biopsy. These patients were not different than patients without biopsy (Table 1). The median time between biopsy and LSM was 4 years, with 25% being performed within the previous year. Median biopsy length was 16 mm. Liver biopsy used as an imperfect gold standard confirmed the diagnostic value of LSM [AUROC = 0.66 (0.60–0.71)] and FT [AUROC = 0.75 (0.70–0.79)] for predicting advanced fibrosis. (Table 3)

**Table 3:** Validation of the proof of concept using liver biopsy: manufacturers' risk factors of false positive/negative are associated with strength of concordance between Elastography and Biopsy.

Characteristics (number patients)	AUROC		
	Liver Stiffness	FibroTest	
	Advanced versus non advanced fibrosis Mean (95% CI) Significance		Significance FT vs stiffness
**All patients (391)**	**0.66 (0.60–0.71)**	**0.75 (0.70–0.79)**	**P = 0.007**
**Manufacturer risk factors**	**P = 0.008**	**P = 0.83**	
Yes (125)	0.56 (0.45–0.65)	0.76 (0.65–0.83)	P = 0.002
None (266)	0.72 (0.65–0.78)	0.74 (0.68–0.80)	P = 0.54
**High risk Elastography**	P = 0.003	P = 0.39	
Yes (111)	0.54 (0.42–0.64)	0.78 (0.68–0.86)	P = 0.0002
No (280)	0.73 (0.66–0.78)	0.73 (0.67–0.79)	P = 0.80
**High risk FibroTest**	P = 0.01	P = 0.03	
Yes (16)	0.88 (0.53–0.98)	0.63 (0.23–0.84)	P = 0.13
No (375)	0.65 (0.59–0.70)	0.76 (0.70–0.80)	P = 0.0004

Patients with recommended criteria had a significantly higher LSM accuracy [n = 266 AUROC = 0.72 (0.65–0.78) ] than patients without these criteria [n = 125; AUROC = 0.54 (0.42–0.64); P = 0.008)]. FT had the same significant diagnostic accuracy among patients with or without LSM recommended criteria [AUROC = 0.79 vs 0.72]. The same results were obtained using the other statistical methods (Supporting Table S4).

### Concordance analysis among patients with recommended criteria

**Figure 2:**
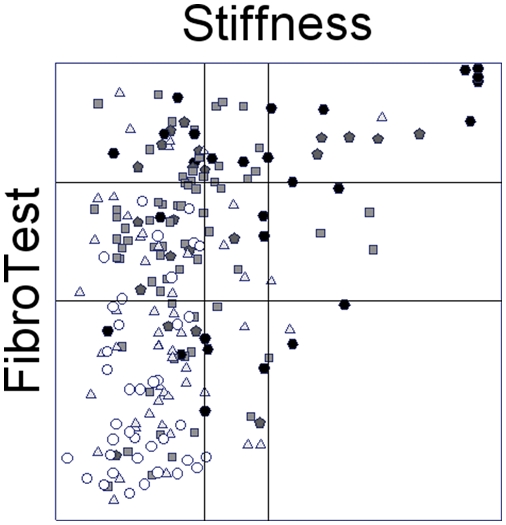
Associations between three estimates of liver fibrosis: FibroTest, liver stiffness measurements and fibrosis stage at biopsy, in 266 patients with recommended criteria. Each point represents the result of biopsy stage using METAVIR scoring system: white circle F0 (no fibrosis), light grey triangle F1 (portal fibrosis), grey square F2 (few septa), light black pentagon F3 (many septa) and dark black hexagon F4 (cirrhosis). These points are distributed according to FibroTest value on the vertical axis and liver stiffness measurements on horizontal axis. The first horizontal line is the FibroTest cutoff (0.48) for stage F2, the second (0.73) for F4. The first vertical line is the stiffness cutoff (8.8 kPa) for stage F2, the second (14.5 kPa) for F4.

Concordances between LSM, FT and biopsy in patients with recommended criteria (n = 266) are detailed in Figure 2 and in supporting Text S2.

LSM and FT, using biopsy as a reference, had similar accuracy with a trend in favor of FT: AUROC (0.72 vs 0.79;P = 0.12), kappa in 2 classes (0.22 vs 0.37; P = 0.03). The mean fibrosis stage presumed using biopsy (1.7; 95%CI 1.6–1.9) was higher than the mean presumed with LSM using 8.8 kPa cutoff (1.4; 95%CI 1.3–1.6; P = 0.0008), but not different than the mean presumed with LSM using 7.1 kPa cutoffs (1.7;1.5–1.8;P = 0.68) and lower than those presumed with FT (2.0;1.8–2.2; P = 0.004).

### Factors associated with strength of concordance

A total of 57 patients with biopsy were assessed with the previously identified LSM high-risk operator; there was no difference in LSM accuracy vs the other operators. The IQR/LSM was 1.5, no different than in the other operators (1.4) but lower than that observed among the 375 patients (with or without biopsy) analyzed by this operator (3.0).

The following factors were associated (not significantly) with lower accuracy of LSM vs FT: BMI>27 kg/m2 (P = 0.04), abdominal fold >30 mm (P = 0.06), and thoracic fold >15 mm (P = 0.08).

### Analyses and validation of discordance cases with presumed cirrhosis (Supporting Table S5)

Among the 53 patients with non-advanced fibrosis with LSM and presumed cirrhosis with FT the failure was attributed to LSM (false negative) in 29 cases, to FT (false positive) in four. Among the 17 patients with presumed cirrhosis using LSM and non-advanced fibrosis using FT, the failure was attributed to LSM in two cases and to FT in seven.

### Evaluation of accuracy, in the absence of a gold standard

The best global model with coherent estimates of tests' accuracy for the diagnosis of advanced fibrosis was the model using the 8.8 kPa cutoff for LSM. There was no significant differences between expected and observed specificities and sensitivities (3 degree of freedom, deviance = 3; P = 0.44). The estimated specificities were for FT 100% and LSM 96%, and sensitivities were for FT 86% and LSM 48%. For the cutoff 7.1 kPa, the model fit also but less well (3 degree of freedom, deviance = 6; P = 0.10). The estimated specificities were for FT 100% and LSM 81% and sensitivities were FT 87% and LSM 67% (Supporting Text S3).

### Sensitivity analyses of manufacturers' recommendations (Supporting Table S6)

The decreases of cutoffs significantly worsen the concordance strength, in all methods.

The increase of success rate to 100% (versus 60%) increased concordance rates but decreased applicability by 50%. Increasing the cutoff of IQR/LSM at 20% instead of 30% did not increase the strength of concordance when the high-risk operator had been excluded.

### Fibrosis, activity and steatosis (Supporting Text S4)

Among the 343 patients without fibrosis using FT, LSM was associated with ST (R = 0.23; P = 0.00001), still significant after adjustment for AT (P = 0.01) and with AT (P<0.00001), still significant after adjustment for ST (P = 0.003). There were significant steatosis and activity effects without interaction. The median LSM was 0.9 kPa higher in patients with presumed steatosis and 1.1 kPa higher in patients with presumed activity vs patients without.

When previous biopsy results were used, (Supporting Table S7) LSM and FT were able to diagnose fibrosis, regardless of the presence of steatosis or activity. Similar steatosis and activity effects on LSM were observed as with ST and AT.

### Confounding variables

Details are given in supporting Text S5. As expected, LSM and FT were highly associated with known risk factors of fibrosis, which could also be variability factors of LSM. LSM, but not FT, was associated with weight (P<0.000001) and BMI (P = 0.000002).

We observed a high association between IQR/LSM and LSM, R = 0.70. Among patients with advanced fibrosis 30% had a non-recommended dispersion of LSM (IQR/LSM>30%), which was twice that in patients without advanced fibrosis: 15% (P<0.0001).

Older age was significantly associated with lower concordance and this could be related to the following more rational factors that were also significantly associated with age: NAFLD, thoracic fold, waist circumference, BMI, serum glucose, ST, and AT.

In patients without fibrosis using FT, the LSM was higher in male (6.0 kPa) than in female (5.2 kPa), but there were also a significant gender differences for confounding factors: BMI and for ST.

## Discussion

The aim of this study was not to validate the diagnostic value of LSM or FT, which have been extensively assessed in numerous studies and meta-analyses. The aim was to describe an original method for assessing imperfect gold standards' variability, in order to reduce the risk of false positive and false negative in the diagnosis of liver fibrosis.

This methodology enabled the recommendations of manufacturers to be validated. These recommendations must be strictly followed as inclusion of patients not adhering to the recommended cutoffs significantly reduces the strength of concordance.

Among the patients satisfying recommendations, the methodology identified a significant LSM operator effect and several variability factors, which had been previously suspected increasing the variability of LSM, using biopsy as a gold standard. In the present study, the retrospective analysis of previous biopsy results confirmed both the proof of concept and the identified variability factors.

### Limitations of the study

A major limitation of the present study is the absence of prospective biopsies in all patients, done the same day as LSM and FT. We used retrospective liver biopsies as an “imperfect gold standard”. The sample size of patients with liver biopsy was much smaller in comparison with the population with non-invasive markers, and only 25% were performed within the year of LSM. However no systematic bias was identified and the characteristics of the two populations with or without liver biopsy were similar. We acknowledge that, for biomarkers of activity (AT) and steatosis (ST), the elapsed time between biopsy and LSM could be another significant variability factor. However, for fibrosis stages, the risk of very significant changes was small. Simultaneous liver biopsy cannot be obtained in such large populations, without major bias.

We used multiple methods and multiple tests, but we used conservative rules to reduce the overall risk of false positive conclusions. The proof of concept analysis and the main variability factors for LSM and FT were highly significant at a P value (P<0.001) lower than the scheduled P value of 0.01 and obtained with at least three different methods. The significance of analyses in patients with biopsy was smaller but in the same directions that in the overall population. The a priori hypothesis and the rational of factors tested are also important to reduce the risk of false positive. The main LSM variability factors identified have both a rational and had been already suspected.

Despite a trend for unifying methods assessing concordance, they are no specific guidelines for choosing in practice one method among the six used in the present study [29].

### Advantages of the study

A major advantage of the present study was the analysis of two tests (LSM and FT) simultaneously performed in a large number of consecutive patients.

### Proof of concept

For the proof of concept, manufacturers' recommendations were used. The methodology implied recommendations being independent between LSM and FT. Indeed there was no relationship between the applicability of FT (mostly related to Gilbert's syndrome, hemolysis and acute sepsis) and the applicability of LSM (number of valid LSM, success rate and IQR/LSM).

### Sensitivity analyses of manufacturers' recommendations

Any change in recommendations can have a direct impact on applicability of LSM, and on the risk of false/positives or negatives. In the present study LSM was applicable in only 70% of consecutive patients. The results strongly suggest that it would be hazardous to decrease the cutoffs of LSM applicability for the diagnosis of advanced fibrosis as suggested by others [15]. Kettaneh et al suggested that for cirrhosis diagnosis only, five valid shots could be sufficient, but the impact of IQR/LSM and success rate had not been assessed [14].

### Identifications of variability factors

An operator effect was identified despite experience of over 100 LSM. Interestingly this operator had a significantly higher IQR/LSM than other operators. Therefore concordance analyses between LSM and FT could be useful to “certify” the operators.

The previously suspected LSM variability factors were also identified by the concordance analyses: older age [14], [30] male gender [14], [19], [30], [31], NAFLD as a cause of chronic liver disease [14], [30], overweight [14], [19], [30], BMI [14], [19], [30], abdominal fold [14], [30], and thoracic fold [31]. The methodology can be used only if a variability factor is associated with only one of the two imperfect gold standards. The main significant factors associated with LSM variability, operator factor, NAFLD, overweight, BMI, abdominal fold, and thoracic fold were not associated with FT. These factors were not associated with FT variability [32]. The identification of factors associated with the variability of LSM and FT is complex as most of them were also factors known to be associated with fibrosis progression.

As with others, we observed a very significant association between LSM and age, which almost disappeared after adjustment with metabolic factors. There is no rationale for a direct impact of age on LSM.

Male gender has already been suspected of being a cause of LSM increase [19], [30], [31]. Confounding variables such as BMI, weight, age and other fibrosis estimates have not been excluded. In the present study we also found an LSM increase (0.8 kPa) in male adjusted on fibrosis, but BMI and steatosis could still explain the difference.

Steatosis is associated with fibrosis and therefore steatosis is at least indirectly associated with LSM [30], [34]. Few studies have assessed whether steatosis was directly associated with LSM independent of fibrosis. Kim et al found on a small number of patients, no significant association between LSM and steatosis [35]. Fraquelli et al observed that the LSM reproducibility was significantly reduced in patients with steatosis at biopsy. However no patients were excluded for IQR> = 30% in that study, and LSM association with steatosis was not detailed. [34]

The present study confirmed that the presence of steatosis (presumed with ST) was associated with higher presumed fibrosis stage either with FT or LSM. The present results also suggest that the presence of steatosis (independently of fibrosis stage) and related anthropometric factors (waist circumference, abdominal fold), could be associated with false positive of LSM in patients without cirrhosis. This is in accordance with the false positive rate of LSM observed by others [35] in patients with steatosis, using biopsy as an endpoint. The rational of a risk of false positive could be an increase of the LSM related to an increase of hepatocytes' stiffness due to triglycerides droplets.

The present study confirmed that overweight and BMI were associated with less applicability of LSM [19], [33], [36] In overweight or obese patients, the fatty thoracic belt attenuates both elastic waves and ultrasound rendering liver stiffness measurement impossible, preventing the risk of false measurement [36].

Activity is associated with fibrosis and therefore activity necroinflammatory activity grades are at least indirectly associated with LSM [30], [34]. Few studies have assessed if activity was directly associated with LSM independently of fibrosis. Coco et al observed that LSM was significantly associated with ALT (adjusted for fibrosis and steatosis) and with activity [30]. In the present we observed an association between necroinflammatory activity and LSM, but less consistent than the association observed with steatosis. The rationale for activity is unknown and could be a transient extra cellular matrix, edema or extent of the inflammatory infiltrate of the septa.

Finally from the populations studied, the cutoffs 0.48 for FT and 7.1 kPa for LSM were validated in a global coherent model for the diagnosis of advanced fibrosis, without using imperfect gold standard. The LSM accuracy was better using 7.1 cutoff with sensitivity+specificity = 1.68 versus 1.44 for the 8.8 cutoff. FT had similar and high specificity and sensitivity for both models (sum 1.81 and 1.86). This is the first time that non-invasive biomarkers have been estimated not using biopsy as a gold standard. The classical evaluation using biopsy as a gold standard obtained for LSM 7.1 kPa specificity = 89% and sensitivity = 67% (sum 1.56) and for FT 83% and 62% respectively (sum 1.45) [8], [9], [10]. These differences are probably mostly explained by the variability of liver biopsy, the variability in applying manufacturers' recommendations and the spectrum bias [3], [15], [22], [23], [33].

In conclusion, this study has validated the concept of using the strength of concordance between two non-invasive estimates of liver fibrosis for the identification of factors associated with variability and precautions of use. Manufacturers' recommendations must be strictly followed. There is a need to better define the upper normal limit value of liver stiffness measurements, as well as the choice of a consensual cutoff for advanced fibrosis, because of the risk of false negatives.

## Supporting Information

Table S1 Characteristics
(0.10 MB DOC)

Table S2 Manufacturers recommendations impact
(0.09 MB DOC)

Table S3 Variability factors
(0.14 MB DOC)

Table S4 Recommendations biopsy
(0.07 MB DOC)

Table S5 Discordant cirrhosis
(0.06 MB DOC)

Table S6 Sensitivity analysis
(0.10 MB DOC)

Table S7 Steatosis activity biopsy
(0.09 MB DOC)

Text S1 Methods
(0.04 MB DOC)

Text S2 Concordance with criteria
(0.02 MB DOC)

Text S3 TAGS
(0.06 MB DOC)

Text S4 Steatosis Activity interaction
(0.07 MB DOC)

Text S5 Confounding variables
(0.03 MB DOC)
