# Supplementary material for: Concordance in a World without a Gold Standard: A New Non-Invasive Methodology for Improving Accuracy of Fibrosis Markers
Source: PLoS One. 2008 Dec 4;3(12):e3857. doi: 10.1371/journal.pone.0003857 (PMC2586659; doi:10.1371/journal.pone.0003857)
Supplement: Table S1 — Characteristics (0.10 MB DOC) [file pone.0003857.s001.doc]

**Supplementary Table S1: Characteristics of included patients**

| **Characteristics** | **All patients** | **Not interpretable Elastography according to manufacturer £** | **Not interpretable FibroTest according to manufacturer £** | **Interpretable for both estimates** | **Patients with Biopsy** |
| --- | --- | --- | --- | --- | --- |
| **Number of patients** | **2004** | **604** | **88** | **1338** | **391** |
| Age at serum, years | 51 (13) | 54 (13)**** | 48 (14)* | 50 (13) | 53 (11) |
| Male | 1238 (62%) | 353 (58%)* | 63 (72%) | 822 (61%) | 265 (68%) |
| *Ethnic origin* |  |  |  |  |  |
| Caucasian | 1467 (73%) | 468 (78%) | 63 (72%) | 956 (71.5%) | 289 (73%) |
| Asian | 138 (7%) | 37 (6%) | 5 (6%) | 100 (7.5%) | 138 (5%) |
| North African | 214 (11%) | 57 (9%) | 10 (11%) | 140 (10.5%) | 52 (13%) |
| Other African | 185 (9%) | 42 (7%) | 10 (11%) | 142 (10.5%) | 30 (8%) |
| *Anthropometric data*$ |  |  |  |  |  |
| Height m | 1,7 (0.1) | 1.7 (0.1) | 1.7 (0.1) | 1.7 (0.1) | 1.7 (0.1) |
| Weight kg | 71 (15) | 76 (17)**** | 71 (14) | 70 (14) | 74 (16) |
| BMI, kg/m2 |  | 26 (5)**** | 24 (4) | 24 (4) | 25 (5) |
| Abdominal fold mm | 23 (13) | 28 (16)**** | 19 (14) | 21 (12) | 24 (14) |
| Thoracic fold mm | 13 (9) | 17 (11)**** | 12 (9) | 12 (7) | 14 (9) |
| Waist circumference cm | 88 (13) | 92 (16)**** | 88 (12) | 86 (12) | 91 (15) |
| *Daily alcohol >=30g/day* | *84 (4%)* | *23 (4%)* | *4 (5%)* | *58 (4%)* | *14 (4%)* |
| ***Diagnosis*** |  |  |  |  |  |
| **Acute disease** | 7 (0.4%) | 1 (0%) | 7 (8%) | 0 (0%) | 0 (0%) |
| **Chronic disease** | 1997 (99.6%) | 603 (100%) | 81 (92%) | 100% | 391 (100%) |
| HCV | 708 (35%) | 181 (30%) | 14 (16%)* | 517 (39%) | 145 (37%) |
| HBV | 363 (18%) | 101 (17%) | 13 (15%) | 255 (19%) | 48 (12%) |
| NAFLD | 296 (15%) | 136 (23%)* | 11 (13%) | 168 (13%) | 66 (17%) |
| HIV coinfection | 172 (9%) | 42 (7%) | 23 (26%)* | 112 (8%) | 53 (14%) |
| ALD | 48 (2%) | 15 (2%) | 1 (1%) | 32 (2%) | 9 (2%) |
| Other | 168 (6%) | 51 (8%) | 3 (3%) | 99 (7%) | 24 (6%) |
| Unknown | 242 (12%) | 77 (13%) | 16 (18%) | 155 (12%) | 46 (12%) |
| **High risk FibroTest** | 88 (4%) | 26 (4%) | 88 (100%) | 0 (0%) | 20 |
| Gilbert | 41 | 3 (1%) | 41 | 0 | 12 (3%) |
| Hemolysis | 31 | 9 (2%) | 31 | 0 | 8 (2%) |
| Acute Inflammation | 8 | 0 (0%) | 8 | 0 | 0 |
| Cholestasis | 5 | 3 (1%) | 5 | 0 | 0 |
| Acute hepatitis | 7 | 1 (0%) | 7 | 0 | 0 |
| **High risk Elastography** | 604 (100%) | 604 (100%) | 26 (30%) | 0 (0%) | 111 (28%) |
| <10 valid measures | 202 | 202 | 5 (6%) | 0 | 38 (10%) |
| Success rate <60% | 262 | 262 | 9 (10%) | 0 | 47 (12%) |
| IQR/LSM >30% | 381 | 381 | 19 (22%) | 0 | 72 (18%) |
| ***Biochemistry*** |  |  |  |  |  |
| ALT IU/L | 69 (206) | 63 (212) | 269 (930)* | 71 (204) | 65 (64) |
| AST IU/L | 48 (38) | 44 (28)* | 59 (85) | 50 (41) | 48 (39) |
| Cholesterol mmol/L | 4.7 (1) | 4.7 (1) | 4.5 (1) | 4.7 (1) | 4.6 (1) |
| Glucose mmol/L | 5.3 (1.8) | 5.4 (1.6)* | 5.5 (2) | 5.3 (1.8) | 5.4 (1.3) |
| Triglycerides mmol/L | 1.3 (0.9) | 1.3 (1) | 1.3 (1) | 1.2 (0.8) | 1.3 (1.0) |
| FibroTest | 0.43 (0.27) | 0.45 (0.27) | 0.66 (0.2)**** | 0.43 (0.27) | 0.51 (0.25) |
| ActiTest | 0.34 (0.25) | 0.32 (0.24)* | 0.41 (0.28)* | 0.35 (0.26) | 0.38 (0.25) |
| SteatoTest | 0.37 (0.23) | 0.41 (0.24)**** | 0.32 (0.22) | 0.35 (0.23) | 0.40 (0.23) |

£ statistical comparison versus patients with low risk $ 1674 patients had anthropometric measurements

Data are mean (SD) or proportion. AST=aspartate aminotransferase. ALT=alanine aminotransferase. GGT= glutamyl transpeptidase. ApoA1=apolipoprotein A1.
