# Supplementary material for: Concordance in a World without a Gold Standard: A New Non-Invasive Methodology for Improving Accuracy of Fibrosis Markers
Source: PLoS One. 2008 Dec 4;3(12):e3857. doi: 10.1371/journal.pone.0003857 (PMC2586659; doi:10.1371/journal.pone.0003857)
Supplement: Table S2 — Manufacturers recommendations impact (0.09 MB DOC) [file pone.0003857.s002.doc]

**Supporting Table S2: Proof of concept: manufacturer risk factors of false positives/negatives are associated with strength of concordance between FibroTest and elastography.**

|  | **Method assessing** | **concordance** |  |  |  |  |
| --- | --- | --- | --- | --- | --- | --- |
| **Characteristics (number patients)** | **AUROC*** | **Kappa 2** | **Kappa 3** | **Spearman** | **Intra Class Coefficient** | **Curve fitting** |
|  | Advanced versus non advanced fibrosis Mean (95% CI) Significance | Advanced versus non advanced fibrosis Kappa M (se) | F0F1 vs F2F3 vs F4 M (se) | FT vs FS Spearman Mean (95% CI) |  | Curbe inequality F-test R2 Linear-Linear |
| **All patients (2004)** | 0.72 (0.70-0.75) | 0.38 (0.02) | 0.29 (0.02) | 0.44 (0.41-0.48) | 0.46 (0.42-0.49) | 0.21 |
| **Manufacturer factors** |  |  |  |  |  |  |
| **High risk Elastography** | P<0.0001 | P=0.08 | P=0.003 | P=0.001 | P<0.05 | P<0.00001 |
| **Yes (**604) | 0.63 (0.58-0.68) | 0.32 (0.04) | 0.21 (0.03) | 0.26 (0.19-0.33) | -0.04 (-0.12-0.04) | 0.10 |
| **No (**1400) | 0.78 (0.75-0.80) | 0.40 (0.025) | 0.32 (0.02) | 0.54 (0.51-0.58) | 0.47 (0.43-0.51) | 0.33 |
| Less than 10 valid measures | P<0.0001 | P=0.0001 | P=0.0002 | P<0.0001 | P<0.05 | P<0.00001 |
| Yes (202) | 0.48 (0.40-0.55) | 0.16 (0.05) | 0.08 (0.04) | -0.09 (-0.23-0.05) | 0.09 (-0.05-0.22) | 0.13 |
| No (1802) | 0.77 (0.74-0.79) | 0.41 (0.02) | 0.31 (0.02) | 0.53 (0.49-0.56) | 0.46 (0.42-0.50) | 0.31 |
| Success rate <60% | P<0.0001 | P=0.0001 | P=0.0001 | P<0.0001 | P<0.05 | P<0.00001 |
| Yes (262) | 0.51 (0.44-0.58) | 0.20 (0.05) | 0.11 (0.04) | 0.01 (-0.12-0.13) | 0.01 (-0.11-0.13) | 0.14 |
| No (1742) | 0.77 (0.74-0.79) | 0.41 (0.02) | 0.32 (0.02) | 0.53 (0.50-0.56) | 0.47 (0.43-0.51) | 0.31 |
| IQR >30% | P=0.60 | P=0.68 | P=0.51 | P=0.50 | NS | P=0.009 |
| Yes (381) | 0.71 (0.65-0.76) | 0.36 (0.05) | 0.26 (0.04) | 0.44 (0.36-0.52) | 0.46 (0.38-0.54) | 0.21 |
| No (1623) | 0.73 (0.70-0.75) | 0.38 (0.02) | 0.29 (0.02) | 0.45 (0.41-0.49) | 0.47 (0.43-0.51) | 0.22 |
| **High risk FibroTest** | P=0.09 | P=0.01 | P=0.09 | NS | NS | P<0.00001 |
| **Yes (**88) | 0.68 (0.55-0.78) | 0.15 (0.07) | 0.16 (0.06) | 0.30 (0.10-0.48) | 0.23 (0.02-0.41) | 0.11 |
| **No (**1916) | 0.78 (0.75-0.80) | 0.39 (0.02) | 0.29 (0.016) | 0.44 (0.41-0.48) | 0.46 (0.37-0.45) | 0.22 |
| Gilbert | P=0.56 | P=0.77 | P=0.86 | NS | NS | P=0.00008 |
| Yes (41) | 0.68 (0.47-0.81) | 0.34 (0.13) | 0.31 (0.09) | 0.38 (0.09-0.62) | 0.31 (0.01-0.56) | 0.15 |
| No (1963) | 0.73 (0.70-0.75) | 0.38 (0.02) | 0.29 (0.02) | 0.44 (0.41-0.48) | 0.46 (0.42-0.49) | 0.22 |
| Hemolysis | P=0.40 | P=0.02 | P=0.02 | NS | NS | P<0.00001 |
| Yes (31) | 0.68 (0.38-0.85) | 0.01 (0.05) | 0.01 (0.05) | 0.29 (-0.07-0.58) | 0.14 (-0.23-0.47) | 0.15 |
| No (1973) | 0.78 (0.76-0.80) | 0.39 (0.02) | 0.30 (0.02) | 0.44 (0.40-0.47) | 0.46 (0.42-0.49) | 0.22 |
| **FT as endpoint** | **P<0.0001** | **P=0.01** | **P=0.04** | **P=0.001** | **P<0.05** | P<0.00001 |
| **Manufacturer risk (666)** | **0.63 (0.59-0.67)** | **0.29 (0.04)** | **0.20 (0.03)** | **0.27 (0.20-0.34)** | **0.19 (0.12-0.26)** | **0.09** |
| **None (1338)** | **0.78 (0.76-0.81)** | **0.42 (0.03)** | **0.33 (0.02)** | **0.55 (0.51-0.59)** | **0.52 (0.48-0.56)** | **0.34** |
| **LSM as endpoint** | **P=0.0001** |  |  |  |  |  |
| **Manufacturer risk (666)** | **0.71 (0.67-0.75)** |  |  |  |  |  |
| **None (1338)** | **0.81 (0.78-0.84)** |  |  |  |  |  |

* FT as enpoint for LSM AUROCs, LSM as endpoint for FT AUROCs

£ statistical comparison versus patients with low risk

HIV * (0.01) vs NAFLD

** When an interaction existed between gender and anthropometric parameters existed the concordance analysis was stratified according to gender.

$ P=0.0002 S3S4 vs S0, $$ P=0.02 S3S4 vs S1

£ P=0.005 NAFLD vs Unknown; P=0.04 NAFLD vs HCV; P=0.01 NAFLD vs HBV; P=0.07 NAFLD vs HIV

££ P=0.0009 NAFLD vs Unknown; P=0.02 NAFLD vs HCV; P=0.006 NAFLD vs HBV; P=0.03 NAFLD vs HIV;

€ P=0.046 S0 vs S2 and vs S3S4

€€ P=0.03 S0 vs S2 and P=0.02 S0 vs S3S4;
