# Supplementary material for: Concordance in a World without a Gold Standard: A New Non-Invasive Methodology for Improving Accuracy of Fibrosis Markers
Source: PLoS One. 2008 Dec 4;3(12):e3857. doi: 10.1371/journal.pone.0003857 (PMC2586659; doi:10.1371/journal.pone.0003857)
Supplement: Table S3 — Variability factors (0.14 MB DOC) [file pone.0003857.s003.doc]

**Supporting Table S3: Potential variability factors associated with strength of concordance between FibroTest and elastography, among patients without manufacturers’ risk factors of false positives/negatives.**

|  | **Method assessing** | **concordance** |  |  |  |  |
| --- | --- | --- | --- | --- | --- | --- |
| **Characteristics (number patients)** | **AUROC*** | **Kappa 2** | **Kappa 3** | **Spearman** | **Intra Class Coefficient** | **Curve fitting** |
| **Potential factors among low risk for both elastography and FT** | Advanced versus non advanced fibrosis Mean (95% CI) | Advanced versus non advanced fibrosis | F0F1 vs F2F3 vs F4 | FT vs LSM Spearman Mean (95% CI) | FT vs LSM ICC Mean (95% CI) | Best fit R2 Linear-Linear |
| Operator | P=0.009 | P=0.10 | P=0.04 | P=0.02 | P=0.001 | P=0.003 |
| Discordant Operator (229) | 0.70 (0.63-0.76) | 0.32 (0.07) | 0.25 (0.05) | 0.41 (0.30-0.51) | 0.42 (0.31-0.52) | 0.20 |
| Other Operators (1109) | 0.80 (0.77-0.82) | 0.44 (0.03) | 0.35 (0.02) | 0.57 (0.53-0.61) | 0.61 (0.57-0.65) | 0.38 |
| **Potential factors among low risk for both elastography and FT and concordant operators** | N=1109 |  |  |  |  |  |
| **N=1109** | **0.80 (0.77-0.82)** | **0.44 (0.03)** | **0.35 (0.02)** | **0.57(0.53-0.61)** | **0.61 (0.57-0.65)** | **0.38** |
| Age at serum, years | P=0.0001 | P=0.02 | P=0.001 | P=0.80 | P=0.002 | P<0.0001 |
| >=50 (541) | 0.75 (0.71-0.79) | 0.37 (0.04) | 0.28 (0.03) | 0.53 (0.46-0.59) | 0.54 (0.48-0.60) | 0.31 |
| <50 (568) | 0.86 (0.82-0.89) | 0.50 (0.04) | 0.42 (0.03) | 0.57 (0.51-0.62) | 0.66 (0.61-0.71) | 0.44 |
| Gender | P=0.96 | P=0.90 | P=0.91 | P=0.90 | P=0.99 | P=0.001 |
| Male (686) | 0.79 (0.76-0.82) | 0.43 (0.04) | 0.34 (0.03) | 0.55 (0.49-0.60) | 0.61 (0.56-0.65) | 0.35 |
| Female (423) | 0.79 (0.74-0.84) | 0.44 (0.05) | 0.35 (0.04) | 0.56 (0.49-0.62) | 0.61 (0.55-0.67) | 0.38 |
| *Ethnic origin* | P=0.19 | P=0.04 | P=0.03 | P=0.40 | P=0.10 | P=0.83 |
| Caucasian (786) | 0.80 (0.76-0.83) | 0.45 (0.03) | 0.34 (0.03) | 0.58 (0.53-0.62) | 0.61 (0.56-0.65) | 0.38 |
| Asian (88) | 0.86 (0.75-0.92) | 0.50 (0.10) | 0.46 (0.09) | 0.58 (0.42-0.70) | 0.55 (0.38-0.68) | 0.32 |
| North African (119) | 0.79 (0.69-0.86) | 0.44 (0.08) | 0.38 (0.07) | 0.53 (0.38-0.65) | 0.61 (0.48-0.71) | 0.35 |
| Black African (116) | 0.76 (0.64-0.84) | 0.27 (0.08) | 0.22 (0.07) | 0.51 (0.37-0.63) | 0.49 (0.38-0.62) | 0.28 |
| *Anthropometric data*** |  |  |  |  |  |  |
| Height |  |  |  |  |  |  |
| Female | P=0.59 | P=0.50 | P=0.48 | P=0.60 | P=0.80 | P=0.24 |
| <=1.55 m (70) | 0.75 (0.61-0.84) | 0.31 (0.12 | 0.34 (0.04) | 0.60 (0.43-0.73 | 0.58 (0.40-0.72) | 0.28 |
| >1.55 m (323) | 0.79 (0.72-0.84) | 0.44 (0.05) | 0.25 (0.06 | 0.52 (0.43-0.59) | 0.56 (0.48-0.63) | 0.35 |
| Male | P=0.77 | P=0.90 | P=0.69 | P=0.90 | P=0.60 | P=0.73 |
| <=1.65 m (58) | 0.80 (0.65-0.89) | 0.41 (0.12) | 0.35 (0.09) | 0.51 (0.45-0.56) | 0.46 (0.23-0.64) | 0.27 |
| >1.65 m (571) | 0.78 (0.74-0.82) | 0.41 (0.04) | 0.31 (0.03) | 0.53 (0.46-0.58) | 0.57 (0.37-0.72 | 0.34 |
| Weight |  |  |  |  |  |  |
| Female | P=0.44 | P=0.29 | P=0.03 | P=0.70 | P=0.70 | P=0.11 |
| >=80 kg (35) | 0.72 (0.41-0.88) | 0.25 (0.17) | 0.07 (0.13) | 0.48 (0.18-0.70) | 0.47 (0.16-0.69) | 0.16 |
| <80 kg (358) | 0.80 (0.74-0.84) | 0.43 (0.05) | 0.35 (0.04) | 0.56 (0.48-0.62) | 0.59 (0.52-0.65) | 0.38 |
| Male | P=0.33 | P=0.13 | P=0.02 | P=0.07 | P=0.06 | P=0.0008 |
| >=90 kg (82) | 0.74 (0.60-0.83) | 0.26 (0.11) | 0.14 (0.08) | 0.35 (0.15-0.53) | 0.39 (0.19-0.56) | 0.16 |
| <90 kg (546) | 0.80 (0.76-0.83) | 0.43 (0.04) | 0.34 (0.03) | 0.57 (0.51-0.62) | 0.60 (0.54-0.65) | 0.37 |
| BMI |  |  |  |  |  |  |
| Female | P=0.80 | P=0.45 | P=0.10 | P=0.65 | P=0.65 | P=0.04 |
| <=27 kg/m2 (318) | 0.80 (0.74-0.85) | 0.41 (0.05) | 0.33 (0.04) | 0.55 (0.47-0.62) | 0.58 (0.50-0.65) | 0.37 |
| 27-30 kg/m2 (45) | 0.80 (0.54-0.92) | 0.52 (0.15) | 0.44 (0.12) | 0.61 (0.38-0.77) | 0.65 (0.44-0.79) | 0.50 |
| >30 kg/m2 (30) | 0.80 (0.26-0.96) | 0.36 (0.17) | 0.13 (0.14) | 0.65 (0.38-0.82) | 0.61 (0.32-0.80) | 0.28 |
| Male | P=0.47 | P=0.07 | P=0.02 | P=0.09 | P=0.10 | P=0.02 |
| <=27 kg/m2 (486) | 0.80 (0.75-0.84) | 0.45 (0.04) | 0.36 (0.03) | 0.56 (0.50-0.62) | 0.60 (0.54-0.65) | 0.39 |
| 27-30 kg/m2 (91) | 0.76 (0.64-0.84) | 0.27 (0.09) | 0.23 (0.07) | 0.48 (0.31-0.63) | 0.50 (0.33-0.64) | 0.24 |
| >30 kg/m2 (52) | 0.76 (0.59-0.87) | 0.30 (0.14) | 0.13 (0.11) | 0.34 (0.07-0.56) | 0.38 (0.12-0.59) | 0.16 |
| Abdominal fold | P=0.14 | P=0.07 | P=0.14 | P=0.04 | P=0.10 | P=0.01 |
| >30 mm (136) | 0.73 (0.62-0.81) | 0.29 (0.08) | 0.23 (0.07) | 0.40 (0.25-0.54) | 0.46 (0.32-0.58) | 0.25 |
| <= 30 mm (711) | 0.80 (0.76-0.83) | 0.43 (0.03) | 0.34 (0.03) | 0.59 (0.54-0.64) | 0.61 (0.56-0.65) | 0.40 |
| Thoracic fold | P=0.12 | P=0.32 | P=0.15 | P=0.15 | P=0.15 | P=0.02 |
| >15 mm (230) | 0.72 (0.61-0.81) | 0.34 (0.09) | 0.24 (0.07) | 0.46 (0.31-0.59) | 0.52 (0.42-0.61) | 0.29 |
| <=15 mm (635) | 0.80 (0.77-0.84) | 0.43 (0.04) | 0.34 (0.03) | 0.59 (0.54-0.64) | 0.61 (0.56-0.66) | 0.40 |
| Waist circumference |  |  |  |  |  |  |
| Female | P=0.95 | P=0.50 | P=0.30 | P=0.80 | P=0.80 | P=0.16 |
| >80 cm (91) | 0.81 (0.68-0.89) | 0.49 (0.11) | 0.40 (0.08) | 0.49 (0.31-0.63) | 0.60 (0.45-0.72) | 0.40 |
| <=80 cm (116) | 0.81 (0.68-0.89) | 0.40 (0.08) | 0.29 (0.07) | 0.56 (0.42-0.67) | 0.58 (0.44-0.69) | 0.45 |
| Male | P=0.03 | P=0.003 | P=0.002 | P=0.02 | P=0.001 | P=0.03 |
| >95 cm (83) | 0.65 (0.52-0.76) | 0.19 (0.10) | 0.14 (0.08) | 0.27 (0.06-0.46) | 0.32 (0.11-0.50) | 0.17 |
| <=95 cm (256) | 0.80 (0.74-0.85) | 0.44 (0.06) | 0.36 (0.04) | 0.59 (0.50-0.67) | 0.64 (0.56-0.71) | 0.44 |
| Daily alcohol >=30g/day | P=0.84 | P=0.85 | P=0.67 | P=0.80 | P=0.50 | P=0.70 |
| Yes (52) | 0.81 (0.65-0.90) | 0.42 (0.14) | 0.39 (0.10) | 0.61 (0.40-0.75) | 0.66 (0.47-0.79) | 0.46 |
| No (1057) | 0.80 (0.77-0.82) | 0.44 (0.03) | 0.35 (0.02) | 0.57 (0.52-0.61) | 0.60 (0.56-0.65) | 0.37 |
| ***Diagnosis*** |  |  |  |  |  |  |
| **Chronic disease cause** | P=0.007 HIV vs NAFLD | P=0.005£ | P=0.0009££ | P=0.02 NAFLD§ | P<0.05 | P<0.0001 |
| HCV (466) | 0.77 (0.72-0.80) | 0.40 (0.04) | 0.30 (0.03) | 0.56 (0.50-0.62) | 0.58 (0.51-0.64) | 0.35 |
| HBV (223) | 0.79 (0.70-0.86) | 0.48 (0.06) | 0.40 (0.06) | 0.49 (0.38-0.58) | 0.53 (0.43-0.62) | 0.31 |
| NAFLD (133) | 0.73 (0.62-0.81) | 0.24 (0.07) | 0.16 (0.06) | 0.32 (0.18-0.45) | 0.39 (0.24-0.53) | 0.15 |
| HIV (99) | 0.88 (0.80-0.94) | 0.45 (0.09) | 0.37 (0.07) | 0.75 (0.65-0.82) | 0.70 (0.58-0.79) | 0.56 |
| ALD (31) | 0.77 (0.53-0.90) | 0.25 (0.17) | 0.31 (0.12) | 0.62 (0.34-0.80) | 0.64 (0.37-0.81) | 0.44 |
| Other (91) | 0.84 (0.67-0.93) | 0.33 (0.13) | 0.21 (0.10) | 0.64 (0.46-0.77) | 0.47 (0.29-0.62) | 0.19 |
| Unknown (67) | 0.86 (0.75-0.93) | 0.62 (0.12) | 0.53 (0.09) | 0.69 (0.54-0.80) | 0.72 (0.58-0.82) | 0.53 |
| **Steatosis** | P=0.006 | P=0.03 | P=0.008 | P=0.50 | P=0.50 | P=0.00002 |
| Yes: S2S3S4 5-100% (133) | 0.88 (0.72-0.85) | 0.59 (0.08) | 0.49 (0.06) | 0.63 (0.51-0.72) | 0.66 (0.55-0.75) | 0.53 |
| No: S0S1 0-5% (620) | 0.76 (0.70-0.80) | 0.38 (0.04) | 0.30 (0.03) | 0.53 (0.48-0.59) | 0.56 (0.50-0.61) | 0.34 |
| **Activity A3** | P=0.68 | P=0.40 | P=0.16 | P=0.90 | P=0.50 | P<0.00001 |
| Yes (190) | 0.78 (0.69-0.84) | 0.31 (0.06) | 0.24 (0.05) | 0.48 (0.37-0.59) | 0.47 (0.35-0.57) | 0.23 |
| No (918) | 0.76 (0.72-0.79) | 0.37 (0.03) | 0.31 (0.02) | 0.48 (0.43-0.53) | 0.55 (0.50-0.59) | 0.31 |
| **Normal ALT 36/25 gender** | P=0.09 | P=0.001 | P=0.05 | P=0.20 | P=0.40 | P<0.00001 |
| Yes (366) | 0.75 (0.69-0.84) | 0.30 (0.05) | 0.27 (0.04) | 0.48 (0.40-056) | 0.55 (0.47-0.62) | 0.31 |
| No (742) | 0.81 (0.78-0.84) | 0.48 (0.03) | 0.37 (0.03) | 0.59 (0.54-0.63) | 0.61 (0.56-0.65) | 0.39 |
| **Normal ALT 45 IU/L** | P=0.23 | P=0.02 | P=0.16 | P=0.38 | P=0.90 | P<0.00001 |
| Yes (579) | 0.77 (0.72-0.81) | 0.35 (0.04) | 0.30 (0.03) | 0.50 (0.44-0.56) | 0.57 (0.51-0.62) | 0.33 |
| No (529) | 0.80 (0.76-0.84) | 0.48 (0.04) | 0.36 (0.03) | 0.57 (0.51-0.63) | 0.59 (0.53-0.64) | 0.36 |

* FT as enpoint for LSM AUROCs, LSM as endpoint for FT AUROCs

£ statistical comparison versus patients with low risk

HIV * (0.01) vs NAFLD

** When an interaction existed between gender and anthropometric parameters existed the concordance analysis was stratified according to gender.

$ P=0.0002 S3S4 vs S0, $$ P=0.02 S3S4 vs S1

£ P=0.005 NAFLD vs Unknown; P=0.04 NAFLD vs HCV; P=0.01 NAFLD vs HBV; P=0.07 NAFLD vs HIV

££ P=0.0009 NAFLD vs Unknown; P=0.02 NAFLD vs HCV; P=0.006 NAFLD vs HBV; P=0.03 NAFLD vs HIV;

§ P=0.02 NAFLD vs HCV and vs Other; P=0.01 NAFLD vs HIV and vs Unknown

P=0.046 S0 vs S2 and vs S3S4

€€ P=0.03 S0 vs S2 and P=0.02 S0 vs S3S4;
