# Supplementary material for: Concordance in a World without a Gold Standard: A New Non-Invasive Methodology for Improving Accuracy of Fibrosis Markers
Source: PLoS One. 2008 Dec 4;3(12):e3857. doi: 10.1371/journal.pone.0003857 (PMC2586659; doi:10.1371/journal.pone.0003857)
Supplement: Table S4 — Recommendations biopsy (0.07 MB DOC) [file pone.0003857.s004.doc]

**Supporting Table S4: Concordance between Fibrosis estimates at biopsy, LSM and FT, stratified on manufacturers recommendations**

|  | **Spearman** Mean (95% CI) |  |  |
| --- | --- | --- | --- |
| Characteristics (number patients) | LSM vs Biopsy | FibroTest vs Biopsy | Significance FT vs LSM |
| **All patients (391)** | 0.33 (0.24-0.42) | 0.47 (0.39-0.54) | P=0.20 |
| **High risk LSM or FibroTest** |  |  |  |
| **Yes (125**) | 0.10 (-0.08-0.27) | 0.46 (0.31-0.59) | P=0.01 |
| **No (266**) | 0.46 (0.36-0.55) | 0.47 (0.37-0.56) | P=0.80 |
| **High risk LSM** | NS | NS |  |
| **Yes (111)** | 0.06 (-0.12-0.24) | 0.51 (0.36-0.64) | P=0.01 |
| **No (280)** | 0.47 (0.37-0.56) | 0.45 (0.35-0.54) | P=0.80 |
| **High risk FibroTest** | NS | NS |  |
| **Yes (**16) | 0.65 (0.23-0.87) | 0.25 (0.28-0.66) | P=0.40 |
| **No (**375) | 0.32 (0.23-0.41) | 0.48 (0.40-0.55) | P=0.05 |

|  | **Method assessing** | **concordance** |  |
| --- | --- | --- | --- |
| **Characteristics (number patients)** | **Kappa 2** |  |  |
|  | FibroScan | FibroTest |  |
|  | Advanced versus non advanced fibrosis Mean (se) Significance | | P Significance FT vs LSM |
| **All patients (391)** | **0.19 (0.05)** | **0.38 (0.05)** | **0.007** |
| **Manufacturer risk factors** | **P=0.25** | **P=0.80** |  |
| **Yes (125)** | **0.11 (0.09)** | **0.40 (0.09)** | **0.02** |
| **None (266)** | **0.22 (0.05)** | **0.37 (0.06)** | **0.06** |
| **High risk LSM** | P<0.05 | NS |  |
| **Yes (**111) | 0.07 (0.09) | 0.44 (0.09) | 0.01 |
| **No (**280) | 0.23 (0.05) | 0.36 (0.06) | 0.08 |
| **High risk FibroTest** | NS | NS |  |
| **Yes (**16) | 0.50 (0.22) | 0.13 (0.20) | 0.03 |
| **No (**375) | 0.17 (0.05) | 0.39 (0.05) | 0.02 |
