# Supplementary material for: Concordance in a World without a Gold Standard: A New Non-Invasive Methodology for Improving Accuracy of Fibrosis Markers
Source: PLoS One. 2008 Dec 4;3(12):e3857. doi: 10.1371/journal.pone.0003857 (PMC2586659; doi:10.1371/journal.pone.0003857)
Supplement: Table S5 — Discordant cirrhosis (0.06 MB DOC) [file pone.0003857.s005.doc]

**Supporting Table S5: Causes of failures among discordant cases for the diagnosis of cirrhosis**

|  | **Possible False negative FS or false positive FT N=53 patients with presumed F4 with FibroTest (>0.74) and F0F1 with elastography (<8.8 kPa)** | | | **Possible false positive FS or false negative FTN=17 patients with presumed F4 with elastography (>14.5 kPa) and F0F1F2 with FibroTest (<0.48)** | | |
| --- | --- | --- | --- | --- | --- | --- |
|  | In favor of FT False negative FS | In favor of FS False positive FT | Indeterminate: endpoint not available or with low negative predictive value | In favor of FT False Positive FS | In favor of FS False negative FT | Indeterminate |
| **At least one independent endpoint** | **29*** | **4** | **20** | **2** | **7** | **8** |
| Highly attributable (Biopsy) | 22 |  | 0 | 2 | 6 | 0 |
| Fibrosis stage at Biopsy | 22: 5 F4, 7 F3, 10 F2 | 4 F1 | 0 | 2: 2 F1 | 6: 3 F4 1 F3 2 F2 | 0 |
| Possibly Attributable (no biopsy) | 7 | 0 | 0 | 0 | 1 | 0 |
| At least two other endpoints | Platelets, prothrombin, hypertension, dysmorphy, ultrasonography, thoracic fold, repeated FS |  |  |  | dysmorphy, varices grade 2 |  |
| **Other endpoints** |  |  | 10 no suspicion |  |  |  |
|  |  | 1 acute flare up hepatitis, 1 cardiac prosthesis | **6 suspected FS false negative:** 5 repeated FS: 6.1, 7.3, 8.1, 8.7,8.7 kPa; 6 IQR/LSM 20-30%; 4 Thoracic fold >= 15mm; 6 SteatoTest>0.38 |  |  | **3 suspected FS false positive:** 3 Thoracic fold > 15mm, 2 teatoTest>0.38; 3 IQR/LSM 20-30% |
|  |  |  | **5 suspected false positive FT:** 5 hapto low <0.12 |  |  | **5 suspected FT false negative:**1 Hapto 1.82 g/L; 4 ApoA1>2.0 g/L |

* Five of the 29 patients had repeated FS with LSM> 8.6, 7 had SteatoTest>0.38, 7 had thoracic fold >= 15mm and 10 IQR/LSM 20-30%

**Analyses and validation of discordance cases with presumed cirrhosis (Supplementary Table 5)**

Among the 53 patients with non-advanced fibrosis with LSM and presumed cirrhosis with FT the failure was attributed to LSM (false negative) in 29 cases, (highly attributable in 22, moderately in 7); failure was attributed to FT (false positive) in four (highly attributable in all: biopsy and one known cause of false positive: 2 hemolysis, one acute flare-up of auto immune hepatitis and one cholestatic flare-up after transplantation). The cause was undetermined in 20 patients. However, a false negative of LSM was suspected in six cases (thickness of thoracic fold and borderline platelets and prothrombin time), and a false positive of FT was suspected in another four (3 low haptoglobin and one elevated GGT).

Among the 17 patients with presumed cirrhosis using LSM and non-advanced fibrosis using FT, the failure was attributed to LSM in two cases (all highly attributable) and to FT in seven (all highly attributable). The cause of failure remained undetermined in eight patients. However, a false positive of LSM was suspected in three patients with elevated thoracic fold thickness, steatosis or IQR/LSM>20%.
