# Supplementary material for: Concordance in a World without a Gold Standard: A New Non-Invasive Methodology for Improving Accuracy of Fibrosis Markers
Source: PLoS One. 2008 Dec 4;3(12):e3857. doi: 10.1371/journal.pone.0003857 (PMC2586659; doi:10.1371/journal.pone.0003857)
Supplement: Table S6 — Sensitivity analysis (0.10 MB DOC) [file pone.0003857.s006.doc]

**Supplementary Table S6: Sensitivity analysis of elastography manufacturer recommended cutoffs**

For LSM the following cutoffs were tested against the recommended cutoffs: for the number of valid measurements, 3 to 9 and 0 to 2 against recommended 10; for success rate 30% to 59% and 0% to 29% against the recommended cutoffs of 100% and 60-99%; for IQR/LSM 30%-40% and greater than 40% against the recommended cutoffs 20-30%, 10-20% and <10%.

|  | **Method assessing** | **concordance** |  |  |  |  |
| --- | --- | --- | --- | --- | --- | --- |
| **Characteristics (number patients)** | **AUROC** | **Kappa 2** | **Kappa 3** | **Spearman** | **Intra Class Coefficient** | **Curve fitting** |
|  | Advanced versus non advanced fibrosis | Advanced versus non advanced fibrosis | F0F1 vs F2F3 vs F4 | FT vs FS Spearman, ICC |  | Best fit R2 Linear-Linear |
| **Manufacturer factors among the 2004 preincluded patients** | Significance N mean (95% CI) | Kappa (se) | Kappa (se) |  |  |  |
|  |  |  |  |  |  |  |
| **Number Valid measurements** | **P<0.0001*** | **P<0.001** | **P<0.001** | **P<0.05** | **P<0.05** | **P<0.0001** |
| ***Recommended >=10 (1802)*** | ***0.77 (0.74-0.79)*** | ***0.41 (0.02)*** | ***0.31 (0.02)*** | ***0.53 (0.49-0.56)*** | ***0.46 (0.42-0.50)*** | ***0.30*** |
| >10 (155) | 0.79 (0.71-0.85) | 0.34 (0.08) | 0.23 (0.06) | 0.56 (0.45-0.66) | 0.57 (0.45-0.67) | 0.35 |
| 10 (1647) | 0.77 (0.74-0.79) | 0.41 (0.02) | 0.32 (0.02) | 0.52 (0.49-0.56) | 0.48 (0.44-0.52) | 0.30 |
| ***Not recommended (202)*** | ***0.48 (0.40-0.55)*** | ***0.16 (0.05)*** | ***0.08 (0.04)*** | ***-0.09 (-0.23-0.05)*** | ***0.09 (-0.05-0.23)*** | ***0.13*** |
| 3 to 9 (62) | 0.66 (0.50-0.78) | 0.33 (0.11) | 0.16 (0.09) | 0.25 (0.00-0.47) | 0.26 (0.01-0.48) | 0.07 |
| 0 to 2 (140) | 0.42 (0.34-0.50) | 0.09 (0.05) | 0.04 (0.04) | -0.20 (-0.03 -0.34) | -0.07 (-0.23-0.10) | 0.18 |
|  |  |  |  |  |  |  |
| **Success rate** | **P<0.001** | **P<0.001** | **P<0.001** | **P<0.05** | **P<0.05** | **P<0.0001** |
| ***Recommended (1742)*** | ***0.77 (0.74-0.79)*** | ***0.41 (0.02)*** | ***0.32 (0.02)*** | ***0.53 (0.50-0.56)*** | ***0.47 (0.43-0.51)*** | ***0.31*** |
| 100% (942) | 0.79 (0.76-0.82) | 0.44 (0.03) | 0.35 (0.02) | 0.57 (0.53-0.62) | 0.58 (0.54-0.62) | 0.35 |
| 60-99% (800) | 0.74 (0.71-0.78) | 0.37 (0.03) | 0.27 (0.03) | 0.48 (0.42-0.53) | 0.51 (0.46-0.56) | 0.26 |
| ***Not recommended (262)*** | ***0.51 (0.44-0.58)*** | ***0.20 (0.05)*** | ***0.11 (0.04)*** | ***0.01 (-0.12-0.13)*** | ***0.01 (-0.11-0.13)*** | ***0.14*** |
| 30-59% (101) | 0.70 (0.58-0.79) | 0.34 (0.09) | 0.20 (0.07) | 0.37 (0.18-0.52) | 0.43 (0.26-0.58) | 0.18 |
| 0-29% (161) | 0.47 (0.38-0.54) | 0.14 (0.06) | 0.06 (0.04) | -0.11 (-0.26-0.05) | -0.03 (-0.18-0.13) | 0.11 |
|  |  |  |  |  |  |  |
| **IQR/median LSM** | **P<0.0001** | **P<0.001** | **P<0.001** | **P<0.05** | **P<0.05** | **P<0.0001** |
| ***Recommended <30% (1623)*** | ***0.73 (0.70-0.75)*** | ***0.38 (0.02)*** | ***0.29 (0.02)*** | ***0.45 (0.41-0.49)*** | ***0.47 (0.43-0.51)*** | ***0.22 (P=0.01)*** |
| <10% (432) | 0.63 (0.57-0.68) | 0.27 (0.04) | 0.23 (0.03) | 0.27 (0.18-0.36) | 0.21 (0.12-0.30) | 0.13 P<0.0001 |
| 10-20% (741) | 0.78 (0.74-0.81) | 0.41 (0.03) | 0.31 (0.03) | 0.55 (0.49-0.59) | 0.54 (0.49-0.59) | 0.32 |
| 20-30% (450) | 0.78 (0.73-0.82) | 0.44 (0.05) | 0.34 (0.03) | 0.52 (0.44-0.58) | 0.57 (0.50-0.63) | 0.33 |
| ***Not recommended (381)*** | ***0.71 (0.65-0.76)*** | ***0.36 (0.05)*** | ***0.26 (0.04)*** | ***0.44 (0.36-0.52)*** | ***0.46 (0.38-0.54)*** | ***0.21*** |
| 31-40% (208) | 0.76 (0.68-0.82) | 0.47 (0.07) | 0.37 (0.05) | 0.53 (0.42-0.62) | 0.57 (0.47-0.66) | 0.33 ***P=0.01*** |
| >40% (173) | 0.65 (0.56-0.73) | 0.23 (0.08) | 0.13 (0.05) | 0.33 (0.19-0.45) | 0.34 (0.20-0.47) | 0.11 |
| **Among quality patients (1109)** |  |  |  |  |  | **P=0.12** |
| **Number valid measurements** | P=0.97 | NS | NS | NS | NS | P=0.50 |
| >10 (81) | 0.80 (0.69-0.88) | 0.35 (0.11) | 0.23 (0.08) | 0.62 (0.46-0.74) | 0.59 (0.43-0.72) | 0.33 |
| 10 (1028) | 0.80 (0.77-0.83) | 0.45 (0.03) | 0.36 (0.02) | 0.57 (0.52-0.61) | 0.60 (0.56-0.64) | 0.38 |
| **IQR/median LSM** | P=0.36 | NS | NS | NS | NS | P=0.11 |
| <10% (215) | 0.82 (0.76-0.87) | 0.44 (0.06) | 0.38 (0.05) | 0.63 (0.54-0.67) | 0.63 (0.54-0.70) | 0.43 P=0.14 |
| 10-20% (554) | 0.80 (0.75-0.84) | 0.44 (0.04) | 0.34 (0.03) | 0.58 (0.52-0.63) | 0.60 (0.54-0.65) | 0.39 |
| 20-30% (340) | 0.79 (0.73-0.83) | 0.45 (0.05) | 0.34 (0.04) | 0.52 (0.44-0.60) | 0.58 (0.50-0.65) | 0.33 |
| **Success rate** | P=0.08 | NS | NS | NS | NS | P=0.02 |
| 100% (639) | 0.82 (0.78-0.85) | 0.47 (0.04) | 0.37 (0.03) | 0.61 (0.56-0.66) | 0.62 (0.57-0.67) | 0.42 |
| 60-99% (470) | 0.77 (0.72-0.81) | 0.41 (0.04) | 0.34 (0.03) | 0.51 (0.44-0.57) | 0.58 (0.52-0.64) | 0.34 |

*P<0.0001 >10 vs 0to2; P<0.0001 10 vs 0to2; P<0.001 10 or more vs 0 to 2; P=0.004 3 to 9 vs 0 to 2; P=0.13 10 or more vs 3 to 9; P=0.09 >10 vs 3to9, P=0.14 3to9 vs 0to2; P=0.43 >10 vs 10;
