# Supplementary material for: Concordance in a World without a Gold Standard: A New Non-Invasive Methodology for Improving Accuracy of Fibrosis Markers
Source: PLoS One. 2008 Dec 4;3(12):e3857. doi: 10.1371/journal.pone.0003857 (PMC2586659; doi:10.1371/journal.pone.0003857)
Supplement: Table S7 — Steatosis activity biopsy (0.09 MB DOC) [file pone.0003857.s007.doc]

**Supporting Table S7: Impact of Steatosis and activity on LSM and FT assessed with biopsy among the non high-risk population**

| Biopsy groups | No Fibrosis biopsyN median 95%CI | Fibrosis Biopsy | Significance between fibrosis | Significance for steatosis effect | Significance for activity effect |
| --- | --- | --- | --- | --- | --- |
| **All** | **N=88** | **N=105** |  |  |  |
| LSM | 6.1 (5.4-6.4) | 8.0 (7.2-8.8) | <0.00001 |  |  |
| FT | 0.35 (0.38-0.42) | 0.66 (0.60-0.69) | <0.00001 |  |  |
| **No Steatosis** | **N=47** | **N=51** |  |  |  |
| LSM | 6.1 (5.3-6.8) | 6.9 (6.3-7.9) | 0.01 | NS 6.1 vs 6.1 |  |
| FT | 0.38 (0.28-0.46) | 0.60 (0.55-0.68) | <0.00001 | NS 0.38 vs 0.29 |  |
| ***No activity*** | ***N=41*** | ***N=27*** |  |  |  |
| LSM | 5.5 (5.1-6.6) | 7.2 (5.9-8.7) | 0.01 | NS 5.5 vs 6.0 | P=0.09 5.5 vs 6.8 |
| FT | 0.37 (0.19-0.45) | 0.66 (0.47-0.75) | 0.0001 | NS 0.37 vs 0.30 | NS 0.37 vs 0.42 |
| ***Activity*** | ***N=5*** | ***N=22*** |  |  |  |
| LSM | 6.8 (3.0-14.6) | 6.6 (5.3-8.1) | NS | NS 6.8 vs 7.3 | NS 6.6 vs 7.2 |
| FT | 0.42 (0.29-0.75) | 0.59 (0.43-0.64) | NS | NS 0.42 vs 0.53 | NS 0.59 vs 0.66 |
| **Steatosis** | **N=41** | **N=54** |  |  |  |
| LSM | 6.1 (4.6-6.4) | 8.9 (8.0-11.9) | <0.00001 | 0.009 8.9 vs 6.9* |  |
| FT | 0.29 (0.21-0.38) | 0.68 (0.63-0.74) | <0.00001 | 0.08 0.68 vs 0.60 |  |
| ***No activity*** | ***N=34*** | ***N=18*** |  |  |  |
| LSM | 6.0 (4.4-6.3) | 9.4 (6.7-21.3) | 0.0001 | NS 9.4 vs 7.2 | NS 6.0 vs 7.3 |
| FT | 0.30 (0.21-0.42) | 0.65 (0.47-0.71) | 0.0002 | NS 0.65 vs 0.66 | NS 0.30 vs 0.53 |
| ***Activity*** | ***N=2*** | ***N=27*** |  |  |  |
| LSM | 7.3 (0-44) | 8.3 (6.7-10.0) | NS | NS 8.3 vs 6.6 | NS 8.3 vs 9.4 |
| FT | 0.53 (0-1) | 0.72 (0.58-0.77) | NS | NS 0.72 vs 0.59 | NS 0.72 vs 0.65 |
| **No activity** | **N=78** | **N=48** |  |  |  |
| LSM | 5.9 (5.1-6.1) | 7.5 (6.8-8.8) | 0.00001 |  | 0.07 5.9 vs 6.8 |
| FT | 0.33 (0.27-0.43) | 0.66 (0.59-0.69) | <0.00001 |  | 0.08 0.33 vs 0.46 |
| **Activity** | **N=7** | **N=59** |  |  |  |
| LSM | 6.8 (4.4-17.1) | 7.9 (6.6-8.4) | NS |  | NS 7.9 vs 7.5 |
| FT | 0.46 (0.37-0.82) | 0.69 (0.60-0.77) | 0.08 |  | NS 0.69 vs 0.66 |
