# Supplementary material for: Concordance in a World without a Gold Standard: A New Non-Invasive Methodology for Improving Accuracy of Fibrosis Markers
Source: PLoS One. 2008 Dec 4;3(12):e3857. doi: 10.1371/journal.pone.0003857 (PMC2586659; doi:10.1371/journal.pone.0003857)
Supplement: Text S1 — Methods (0.04 MB DOC) [file pone.0003857.s008.doc]

***Supporting Text S1: Definition of the strength of concordance and statistical analysis***

The strength of concordance between LSM and FT was assessed using six methods, three categorical, two quantitative and one mixed.

For the first method we used the area under the receiver operating characteristic curve (AUC). [15-17] The AUC combines the sensitivity (Se) and specificity (Sp) of a given quantitative marker for the diagnosis of a specific definition of fibrosis. The disease was defined as advanced (or bridging) fibrosis. The Se is usually assessed in patients with advanced fibrosis (i.e. stages F2, F3, F4 in the METAVIR scoring system) [18] and the Sp in non-advanced fibrosis (i.e. stages F0, F1). For factors related to elastography the end point was advanced fibrosis as defined using FT (above 0.48). [15] For factors related to FT the end point was advanced fibrosis as defined as LSM above 8.8 kPa. [13,14] The AUC was used as a measurement of discrimination and esti­mated using the empirical (non-parametric) method of DeLong et al. [19]. The paired method of Zhou et al. [20] was used for statistical comparisons of AUCs.

For the second and third methods, we used the kappa reliability test (K) [21] for 2 (K2) fibrosis stages (F2F3F4 vs F0F1) and 3 (K3) fibrosis stages (F0F1, vs F2F3 vs F4), with recommended cutoffs being 8.8 and 14.5kPa for LSM and 0.48, and 0.74 for FT. [13-15]

For the fourth method we used the non-parametric Spearman coefficient of correlation (R). For the analysis of associations between LSM and steatosis presumed with SteatoTest (ST) and activity presumed with ActiTest (AT), adjusted for fibrosis stage as defined using FT cutoffs, we used both R and partial correlation (pR). This enabled LSM to be assessed for an association with presumed steatosis or activity, independently of fibrosis estimate.

For the fifth method, we used the intraclass coefficient of correlation (ICC) [21], between FT (range 0 to 1) and LSM (range 0 to 75) normalized by decimal logarithmic transformation.

For the sixth method, we assessed the best curve fitting between FT and LSM using the regression curve randomization test. A total of 40 models were tested and the linear-linear model was the most appropriate. Logarithmic transformation was used because of a skewed distribution. The null hypothesis was that the all the group curves coincided. Squared coefficient of correlation (R2) was used as an assessment of the strength of association between the FT and LSM best fitting curves. [22] This method also enabled the identification of cutoffs of quantitative factors for which the curves no longer coincided.

The same methods were used to identify other factors possibly associated with variability of each technique.

All analyses were performed with NCSS software (Kaysville, Utah, USA). [22]

The program “TAGS” was used for the evaluation of FT and LSM accuracy, in the absence of a perfect gold standard. [23] The model used two populations comparing FT and LSM, without the perfect disease status, the present data set (n=1,109 patients) and the data published by Castera et al (n=183)[9], and two disease free reference populations (925 blood donors and 429 healthy subjects). [24,25,26] Six parameters were estimated (prevalence of advanced fibrosis in the two populations, sensitivity and specificity of the two tests) with 9 degree of freedoms using the maximum likelihood estimation (Newton Raphson and expectation maximization algorithms). [23]

A sensitivity analysis has been performed using the 7.1 cutoff,[9] also used for advanced fibrosis instead of 8.8 kPa [14].

***Potential factors of variability***

The following potential factors of LSM variability were tested: operator effect, male gender, steatosis (presumed with SteatoTest), necroinflammatory activity (presumed with ActiTest), normal transaminases ALT, anthropometric factors (BMI, abdominal and thoracic folds, waist circumference), cause of liver disease and ethnic origin.

The following potential factors of FT variability were tested: normal transaminases ALT, cause of liver disease and ethnic origin.

***Validation of results using biopsy***

Liver biopsy was used to confirm the results observed with the proposed methods. The accuracies of LSM and FT in the different populations at risk were compared using the same statistical methods.

***Validation of results using discordance in cirrhosis diagnosis***

In order to attribute the cause of discordance between FT and LSM, all the cases with a high level of discordance (cirrhosis as predicted by one method, and F2 or lower stage by the other method) were reviewed to identify an independent endpoint for cirrhosis as previously published [7].

Independent (from FT and LSM) endpoints used for assessing extensive fibrosis and cirrhosis were stage METAVIR F3 or F4 at liver biopsy, a platelet count of 150 000 cells/mL, prothrombin time <70% of control, hyaluronic acid > 100ug/L, obvious cirrhosis on ultrasound (segment IV atrophy, splenomegaly), a reduction or reversal of portal flow, gradient portal pressure > 5 mmHg, and grade 2 or 3 esophageal varices. Repeated assessments of liver injury, either by a subsequent LSM or FT-AT during follow-up, were also considered. Factors identified as being associated with FT or LSM variability (thoracic fold, steatosis) were also assessed among these patients.

Discordance was considered highly attributable to FT failure if (a) the FT result was non advanced fibrosis, whereas the LSM result was cirrhosis in the presence of stage F3 or F4 at biopsy, (b) or at least two other independent signs of cirrhosis. Discordance was considered moderately attributable to FT failure if the FT result was non advanced fibrosis, whereas the LSM result was cirrhosis in the presence of one independent sign of cirrhosis.

Discordance was considered highly attributable to LSM failure if (a) the LSM result was non advanced fibrosis, whereas the FT result was cirrhosis in the presence of stage F3 or F4 at biopsy, (b) of at least two other independent signs of cirrhosis. Discordance was considered moderately attributable to LSM failure if the LSM result was non advanced fibrosis, whereas the FT result was cirrhosis in the presence of one independent sign of cirrhosis

***Biochemical markers***

FibroTest, ActiTest and SteatoTest (Biopredic­tive, Paris, France) were performed according to published recommendations. [25,27]

***Liver stiffness measurements***

Patients were studied using the non-invasive method of transient elastography (Fibroscan, Echosens, Paris, France). The stiffness results are expressed in kilopascals (kPa). The technique was performed trained (more than 100 measurements) senior hepatologists, blinded to all other characteristics, and according to the manufacturers’ recommendations. The following recommended cutoffs were used: 5.1, 8.8, 9.5 and 14.5 kPa for the F0, F1, F2, F3 and F4 staging respectively. [14] We took 8.8 as cutoff for advanced fibrosis (F2 or greater) and not 7.1 kPa [10] as the 95% percentiles of healthy population is 7.8 for female and 8.0 for male [26].

***Biopsy***

Liver biopsy was performed through a percutaneous route, using a 16 gauge Hepafix needle (Braun Malsungen, Germany). Liver samples were paraffin embedded and stained with hematoxylin-eosin-safran and Sirius red. Staging and grading were performed blinded to non-invasive methods, according to METAVIR scoring system and according to Brunt et al for NAFLD. [28].

***Sensitivity analyses of manufacturers’ recommendations***

Sensitivity analyses were performed in order to check, using the same methodology, if the recommended cutoffs were adequate for increasing the strength of concordance between LSM and FT.
