# Supplementary material for: Concordance in a World without a Gold Standard: A New Non-Invasive Methodology for Improving Accuracy of Fibrosis Markers
Source: PLoS One. 2008 Dec 4;3(12):e3857. doi: 10.1371/journal.pone.0003857 (PMC2586659; doi:10.1371/journal.pone.0003857)
Supplement: Text S2 — Concordance with criteria (0.02 MB DOC) [file pone.0003857.s009.doc]

***Supporting File Text S2: Concordance analysis among patients with recommended criteria***

LSM and FT, using biopsy as a reference, had similar accuracy with a trend in favor of FT: AUROC (0.72 vs 0.79;P=0.12), kappa in 2 classes (0.22 vs 0.37; P=0.03), kappa in 3 classes (0.19 vs 0.26 P=0.32), kappa in 5 classes (0.10 vs 0.16; P=0.16). The ICC between stages presumed by the 3 estimates were not different 0.45 between LSM and biopsy and 0.49 between FT and biopsy (P=0.36). The mean fibrosis stage presumed using biopsy (1.7; 95%CI 1.6-1.9) was higher than the mean presumed with LSM using Ketanneh cutoffs (1.4; 95%CI 1.3-1.6; P=0.0008), not different than the mean presumed with LSM using Castera cutoffs (1.7;1.5-1.8;P=0.68) and lower than those presumed with FT (2.0;1.8-2.2; P=0.004).

The Spearman R was 0.47 between FT and biopsy, and 0.46 between LSM and biopsy. When LSM was translated into stages the R decreased to 0.41 using Ketanneh and 0.44 and using Castera cutoffs.
