# Supplementary material for: Concordance in a World without a Gold Standard: A New Non-Invasive Methodology for Improving Accuracy of Fibrosis Markers
Source: PLoS One. 2008 Dec 4;3(12):e3857. doi: 10.1371/journal.pone.0003857 (PMC2586659; doi:10.1371/journal.pone.0003857)
Supplement: Text S3 — TAGS (0.06 MB DOC) [file pone.0003857.s010.doc]

***Supporting Test S3: Modeling evaluation of accuracy in the absence of a gold standard***

The best estimations of tests’ accuracy for the diagnosis of advanced fibrosis were for FT 100% (95%CI 99-100%), 70% (66-75%) and for LSM 96% (95%CI 95-97%) and 67% (95%CI 63%-71%) for specificity and sensitivity respectively. The model fit with a deviance=7 (3 degree of freedom, P=0.07, that is no significant differences between expected and observed specificities and sensitivities) for the cutoff 7.1 kPa defining advanced fibrosis. For the 8.8 kPa cutoff the model does not fit with a significant deviance = 14 (P=0.003). The estimated specificity and sensitivity of LSM were significantly lower 90% and 47% respectively.

Population 1 is the 1109 patients included in the present study. Population 2 is the 183 patients included in the Castera et al study.

**Population 1, 8.8 kPa for F2**

FTF234

STIF234 0 1 Total

0 631 217 848

1 53 208 261

Total 684 425 1109

**Population 1, 7.1 kPa for F2**

FT F234

STIFFNESS 0 1 Total

Up To 7 541 149 690

7 To 80 143 276 419

Total 684 425 1109

**Population 2, 8.8 kPa for F2**

77 30 107

15 61 76

92 91 183

**Population 2, 7.1 kPa for F2**

69 20 89

23 71 94

92 91 183

**Population 3, disease free are blood donors (Ref 25) and from healthy subjects (Ref 26).**

The positive rate was 0% for FT and 2.5% for LSM at 8.8 kPa cut off and 5% at 7.1 kpa cutoff

**Details of results :**

**Cutoff 8.8 kPa**

Results from Rweb

DATA SUMMARY

2 Population(s); 2 Tests; 1 Reference Population(s)

d.d.l: 9 ; parameters: 6

test1 test2 pop1 pop2 RefInd RefInf

1 0 0 631 120 964 0

2 1 0 217 33 0 0

3 0 1 53 10 36 0

4 1 1 208 20 0 0

pre1 pre2 Sp1 Sp2 Se1 Se2

Best Guess 0.5 0.5 0.95 0.9 0.8 0.6

EXPECTATION MAXIMISATION

$Iterations

[1] 560

$LogLikelihood

[1] -1567.608

$Estimations

pre1 pre2 Sp1 Sp2 Se1 Se2

Est 0.3917 0.2961 1 0.9279 0.9783 0.477

NEWTON-RAPHSON

$Iterations

[1] 40

$LogLikelihood

[1] -1556.008

$Estimations

pre1 pre2 Sp1 Sp2 Se1 Se2

Est 0.4428 0.3398 1 0.9637 0.8640 0.4770

CIinf 0.4022 0.2661 NaN 0.9502 0.7871 0.4325

CIsup 0.4842 0.4222 1 0.9737 0.9160 0.5218

WARNING 1: test results are assumed to be independent conditional on infection or disease status

WARNING 2: tests are supposed to have constant sensitivity and specificity in all populations

Expected Results (NR) and Goodness-of-fit test

$Expected

test1 test2 pop1 pop2 RefInd RefInf ExpPop1 ExpPop2 ExpRefInd ExpRefInf

1 0 0 631 120 964 0 630.43 120.85 963.71 0

2 1 0 217 33 0 0 221.90 28.10 0.00 0

3 0 1 53 10 36 0 54.29 8.42 36.29 0

4 1 1 208 20 0 0 202.37 25.63 0.00 0

$Test

Max LogLikelihood: Achievable Obtained Deviance d.f. p value

-1554.643 -1556.008 2.729797 3 0.4351874

$Commentary

[1] "The model could fit"

Residuals correlations between test

$ResCor

Corr1-2

pop 1 : 0.01379382

pop 2 : -0.11389369

$Commentary

[1] "The residuals should be randomly distributed around 0"

**Cutoff 7.1 kPa**

Results from Rweb

DATA SUMMARY

2 Population(s); 2 Tests; 1 Reference Population(s)

d.d.l: 9 ; parameters: 6

test1 test2 pop1 pop2 RefInd RefInf

1 0 0 541 69 874 0

2 1 0 149 20 0 0

3 0 1 143 23 126 0

4 1 1 276 71 0 0

pre1 pre2 Sp1 Sp2 Se1 Se2

Best Guess 0.5 0.5 0.95 0.9 0.8 0.6

EXPECTATION MAXIMISATION

$Iterations

[1] 259

$LogLikelihood

[1] -2011.991

$Estimations

pre1 pre2 Sp1 Sp2 Se1 Se2

Est 0.3832 0.4973 1 0.7646 1 0.6725

NEWTON-RAPHSON

The Matrix is singular : no SE available

$Iterations

[1] 36

$LogLikelihood

[1] -1972.495

$Estimations

pre1 pre2 Sp1 Sp2 Se1 Se2

Est 0.476 0.6148 1 0.8738 0.8058 0.6725

CIinf NaN NaN NaN NaN NaN NaN

CIsup NaN NaN NaN NaN NaN NaN

WARNING 1: test results are assumed to be independent conditional on infection or disease status

WARNING 2: tests are supposed to have constant sensitivity and specificity in all populations

Expected Results (NR) and Goodness-of-fit test

$Expected

test1 test2 pop1 pop2 RefInd RefInf ExpPop1 ExpPop2 ExpRefInd ExpRefInf

1 0 0 541 69 874 0 541.41 68.75 873.84 0

2 1 0 149 20 0 0 139.31 29.69 0.00 0

3 0 1 143 23 126 0 142.25 23.59 126.16 0

4 1 1 276 71 0 0 286.03 60.97 0.00 0

$Test

Max LogLikelihood: Achievable Obtained Deviance d.f. p value

-1969.404 -1972.495 6.181487 3 0.1031067

$Commentary

[1] "The model could fit"

Residuals correlations between test

$ResCor

Corr1-2

pop 1 : -0.02225285

pop 2 : 0.11216235

$Commentary

[1] "The residuals should be randomly distributed around 0"
