# Supplementary material for: Concordance in a World without a Gold Standard: A New Non-Invasive Methodology for Improving Accuracy of Fibrosis Markers
Source: PLoS One. 2008 Dec 4;3(12):e3857. doi: 10.1371/journal.pone.0003857 (PMC2586659; doi:10.1371/journal.pone.0003857)
Supplement: Text S4 — Steatosis Activity interaction (0.07 MB DOC) [file pone.0003857.s011.doc]

***Supporting Text S4: Fibrosis, activity and steatosis***

*Model one using FT cutoffs for fibrosis standardization*

In this first model we analyzed the associations between LSM and ST and AT, in each strata of fibrosis stage defined using FT cutoffs. The partial correlation analysis in each strata, permitted to assess if steatosis or activity were associated with discordance, independently of fibrosis estimates.

F0 strata

In 500 patients presumed F0 with FT, LSM in univariate analysis was associated with FT (R=0.24), ST (R=0.23) and AT (R=0.26) (all P<0.00001). The median LSM was 5.9 kPa (95% CI 5.4-6.7) in 63 patients with steatosis vs 5.0 in 278 patients without steatosis (95%CI 4.8-5.3; P=0.001). The median LSM was 6.3 kPa (95% CI 5.6-7.6) in 37 patients with activity vs 5.0 in 461 patients without activity (95%CI 4.9-5.3; P=0.0002). In 13 patients with steatosis and activity the median LSM was 7.6 (95%CI 4.9-8.9).

Discordant patients (possible FT false negative or FS false positive) (F0 FT and F1F2F3F4 FS n=251) vs concordant (n=248) had higher AT (0.26 95%CI 0.24-0.29 vs 0.17 95%CI 0.16-0.19; P<0.00001) and higher ST (0.38 95%CI 0.34-0.41 vs 0.27 95%CI 0.24-0.30; P=0.00003).

When adjusted on FT (partial correlation) LSM was still associated with ST (R=0.19; P=0.0003), and with AT (R=0.19; P=0.00001).

When only the 343 F0 patients with both AT and ST were analyzed (patients with only FT and AT excluded), similar results were observed. Among the 343 patients without fibrosis at FT, LSM was associated with ST (R=0.23; P=0.00001), still significant after adjustment on AT (pR=0.13; P=0.01) and with AT (R=0.25; P<0.00001), still significant after adjustment on ST (pR=0.16; P=0.003). There were significant steatosis and activity effects without interaction. The median LSM was 0.9 kPa higher in patients with presumed steatosis than without [5.9 kPa (95% CI 5.4-6.7) in 64 patients with steatosis vs 5.0 in 279 patients without steatosis (95%CI 4.8-5.3; P=0.001)]. The median LSM was 1.1 kPa higher in patients with presumed activity [6.4 kPa (95% CI 5.9-8.0) in 23 patients with activity vs 5.3 in 320 patients without activity (95%CI 4.9-5.5; P=0.002)]. The range was 5.0 kPa (4.8-5.3) in 269 patients without steatosis and activity to 7.6 (95%CI 4.9-8.9; P=0.001) in 13 patients with steatosis and activity.

**Table S: Impact of Steatosis and Activity on LSM assessed with biomarkers biopsy among the 343 non high risk patients without Fibrosis (F0) presumed at FT**

|  | **No Steatosis** | **Steatosis** | Significance for steatosis effect |
| --- | --- | --- | --- |
| N | **N=279** | **N=64** | **P=0.001** |
| LSM median (95%CI) | **5.0 (4.8-5.3)** | **5.9 (5.4-6.7)** |  |
|  |  |  |  |
|  |  |  |  |
| ***No activity N=320*** | *N=269* | *N=51* |  |
| **5.3 (4.9-5.5)** | 5.0 (4.8-5.3) | 5.9 (5.3-6.3) | P=0.02 |
| ***Activity N=23*** | *N=10* | *N=13* |  |
| **6.4 (5.9-8.0)** | 6.2 (4.4-6.8) | 7.6 (4.9-8.9) | P=0.15 |
|  |  |  |  |
| **P=0.002** | P=0.12 | P=0.12 | P=0.001 7.6 vs 5.0 |

**Summary in patients with biopsy**

LSM was slightly increased in patients with steatosis versus in those without (median 0.7 kPa P=0.09), but only significantly in patients with fibrosis (median increase of 2 kPa; P=0.009), with no change in patients without fibrosis (median 0 kPa). This interaction was not observed for FT. LSM was increased in patients with versus in those without activity (median 1.5 kPa; P=0.003), without interaction with fibrosis: median 0.9 kPa increase in patients without fibrosis (P=0.07), and 0.4 kPa increase in patients with fibrosis (P=0.80).

F1 strata

In 184 patients presumed F1 with FT, in univariate analysis LSM was not associated with FT (R=0.11) and AT (R=0.02) and only associated with ST (R=0.19 P=0.02). The median LSM was 7.0 kPa (95% CI 6.0-8.1) in 24 patients with steatosis vs 5.9 (95%CI 5.3-6.1) in 118 patients without steatosis (P=0.02). The median LSM was in 25 patients with activity 6.8 kPa (95%CI 5.8-8.5) vs 5.9 kPa (95% CI 5.5-6.3; P=0.11) in 159 patients without activity. When adjusted on FT (partial correlation) LSM was still associated with ST (R=0.21; P=0.01), and not with AT (R=0.02; P=0.82).

F2 strata

In 72 patients presumed F2 with FT, LSM was only associated with ST (R=0.42 P=0.001) and not associated with FT (R=0.07) and AT (R=0.11). The median LSM was 10.1 kPa (95% CI 5.7-38.5) in 7 patients with steatosis vs 6.6 (95%CI 5.5-7.2) in 48 patients without steatosis (P=0.009). The median LSM was 8.3 kPa (95%CI 5.7-10.6) in 20 patients with activity vs 6.6 kPa (95% CI 5.6-7.3; P=0.15) in 52 patients without activity. When adjusted on FT (partial correlation) LSM was still associated with ST (R=0.43; P=0.001), and not with AT (R=0.10; P=0.39).

F3 strata

In 168 patients presumed F3 with FT, LSM was associated with FT (R=0.16 P=0.04), and associated with ST (R=0.40, P=0.0002) and AT (R=0.23, P=0.003). The median LSM was 12.7 kPa (95% CI 6.3-33.0) in 12 patients with steatosis vs 7.1 (95%CI 6.3-8.1) in 99 patients without steatosis (P=0.02). The median LSM was 8.8 kPa (95%CI 7.9-10.5; P=0.21) in 61 patients with activity vs 6.6 kPa (95% CI 6.1-7.7; P=0.0003) in 107 patients without activity. When adjusted on FT (partial correlation) LSM was still associated with ST (R=0.39; P=0.00002), and with AT (R=0.21; P=0.007).

F4 strata

In 184 patients presumed F4 with FT, LSM was associated with FT (R=0.37 P<0.0001), and associated with ST (R=0.33, P=0.008) and not with AT (R=0.02). The median LSM was 19 kPa (95% CI 13-32) in 25 patients with steatosis vs 10 kPa (95%CI 9-12; P=0.001) in 76 patients without steatosis. The median LSM was 11.8 kPa (95% CI 10.1-14) in 119 patients with activity vs 15 kPa (95%CI 10.9-21.3; P=0.21) in 67 patients without activity.

Discordant patients (possible FS false negative or FT false positive) (F4 FT and F0F1F2F3 FS n=102) vs concordant (n=82) had not higher AT (0.62 95%CI 0.57-0.66 vs 0.60 95%CI 0.54-0.65; P=0.59) and lower ST (0.40 95%CI 0.35-0.41 vs 0.53 95%CI 0.46-0.59; P=0.001).

When adjusted on FT (partial correlation) LSM was still associated with ST (R=0.29; P=0.003), and not with AT (R=-0.06; P=0.42).

F0F1 strata

In 683 patients presumed F0F1 with FT, LSM was associated with FT (R=0.28), ST (R=0.23) and AT (R=0.23) (all P<0.00001). When adjusted on FT (partial correlation) LSM was still associated with ST (R=0.20; P<0.00001), and with AT (R=0.15; P=0.00006).

F0F1F2 strata

In 756 patients with F0F1F2 presumed with FT, LSM was associated with FT (R=0.33), ST (R=0.26) and AT (R=0.25) (all P<0.00001). When adjusted on FT (partial correlation) LSM was still associated with ST (R=0.22; P=0.01), and with AT (R=0.15; P=0.00005).

F0F1F2F3 strata

In 756 patients with F0F1F2 presumed with FT, LSM was associated with FT (R=0.33), ST (R=0.26) and AT (R=0.25) (all P<0.00001). When adjusted on FT (partial correlation) LSM was still associated with ST (R=0.22; P=0.01), and with AT (R=0.15; P=0.00005).

F2F3F4 strata

In 426 patients presumed F2F3F4 with FT, LSM was associated with FT (R=0.47), ST (R=0.43) and AT (R=0.27) (all P<0.00001). When adjusted on FT (partial correlation) LSM was still associated with ST (R=0.37; P<0.00001), and with AT (R=0.11; P=0.03).

F3F4 strata

In 344 patients with F3F4 presumed with FT, LSM was highly associated with FT (R=0.46 P<0.00001), with ST (R=0.44, P<0.00001) and with AT (R=0.24, P<0.00001). When adjusted on FT (partial correlation) LSM was still associated with ST (R=0.35; P=0.01), and not with AT (R=0.09; P=0.09).

The strength of concordance between FS and FT was significantly higher in F3F4 (n=354 R=0.46) than in F0F1F2 (n=755 R=0.33 P=0.001, Curve fitting P<0.00001).

*Model two using FS cutoffs for fibrosis standardization*

In this second model we analyzed the associations between LSM and ST and AT, in each strata of fibrosis stage defined using FS cutoffs. The limitation of this model is the absence of rational for liver stiffness increase between F0 and F1 and probably only a small stiffness increase between F1 and F2. Therefore it is possible that the LSM cutoff for F1 (in comparison with F0 at LSM) mainly identified patients with more steatosis or activity and not patients with more fibrosis.

F0 strata

In 360 patients presumed F0 with FS, FT was associated with LSM (R=0.17; P=0.0009), with AT (R=0.48; P<0.0001) and borderline with ST (R=0.12; P=0.052). Discordant patients (possible FS false negative or FT false positive) (F0 FS F1F2F3F4 FT n=165) vs concordant (n=195) had higher AT (0.28 95%CI 0.25-0.31 vs 0.16 95%CI 0.14-0.18; P<0.00001) and no difference between ST. When adjusted on LSM (partial correlation) FT was still associated with AT (R=0.46; P<0.00001), and not with ST (R=0.12; P=0.07).

F1 strata

In 488 patients presumed F1 with FS, FT was associated with LSM (R=0.22; P<0.00001), AT (R=0.43; P<0.00001), and not with ST (R=0.07; P=0.21). Discordant patients (possible FS false negative) (F1 FS vs F2F3F4 FT n=168) vs concordant (n=320) had higher AT (0.45 95%CI 0.42-0.49 vs 0.28 95%CI 0.26-0.30; P<0.0001) and no difference for ST. When adjusted on LSM (partial correlation) FT was still associated with AT (R=0.42; P<0.00001), and not with ST (R=0.04; P=0.50).

F2 strata

In 23 patients presumed F2 with FS, FT was not associated with LSM (R=0.13; P=0.55), associated with AT (R=0.66; P=0.0006) and not with ST (R=0.23; P=0.33). Discordant patients (possible FS false positive) (F2 FS vs F0F1 FT n=9) vs concordant (n=14) had lower AT (0.21 95%CI 0.06-0.35 vs 0.56 95%CI 0.44-0.69 vs; P=0.003) and no difference for ST. When adjusted on LSM (partial correlation) FT was still associated with AT (R=0.66; P=0.0008), and not with ST (R=0.20; P=0.41).

F3 strata

In 120 patients presumed F3 with FS, FT was not associated with LSM (R=0.05; P=0.62), was associated with AT (R=0.53; P<0.00001) and negatively with ST (R=-0.28; P=0.01). Discordant patients (possible FS false positive) (F3 FS vs F0F1F2 FT n=49) vs concordant (n=71) had lower AT (0.35 95%CI 0.27-0.42 vs 0.58 95%CI 0.52-0.64 vs; P<0.00001) and higher ST (0.52 95%CI 0.44-0.59 vs 0.40 95%CI 0.34-0.46 P=0.02). When adjusted on LSM (partial correlation) FT was still associated with AT (R=0.43; P<0.00001), and negatively with ST (R=-0.28; P=0.01).

F4 strata

In 118 patients presumed F4 with FS, FT was associated with LSM (R=0.31 P=0.0008), with AT (R=0.27; P=0.003) and not associated with ST (R=0.03, P=0.79). Discordant patients (possible FS false positive) (n=36) vs concordant (n=82) had lower AT (0.46 95%CI 0.35-0.56 vs 0.60 95%CI 0.54-0.65; P=0.02) and no difference between ST. When adjusted on LSM (partial correlation) FT was still associated with AT (R=0.34; P=0.0002), and not with ST (R=-0.05; P=0.70).

F0F1 strata

In 848 patients presumed F0F1 with FS, FT was associated with LSM (R=0.37; P<0.00001), with AT (R=0.50; P<0.00001) and with ST (R=0.14; P=0.0007). Discordant patients (possible FS false negative or FT false positive) (n=218) vs concordant (n=630) had higher AT (0.43 95%CI 0.40-0.46 vs 0.24 95%CI 0.22-0.25; P<0.0008) and no difference between ST. When adjusted on LSM (partial correlation) FT was still associated with AT (R=0.43; P<0.00001), and not with ST (R=0.07; P=0.09).

F2F3F4 strata

In 261 patients presumed F2F3F4 with FS, FT was associated with LSM (R=0.46; P<0.00001), with AT (R=0.45; P<0.00001) and not with ST (R=0.01; P=0.93). Discordant patients (possible FS false negative or FT false positive) (n=53) vs concordant (n=208) had higher AT (0.57 95%CI 0.54-0.61 vs 0.28 95%CI 0.21-0.34; P<0.00001) and no difference between ST. When adjusted on LSM (partial correlation) FT was still associated with AT (R=0.45; P<0.00001), and not with ST (R=-0.14; P=0.08).

F0F1F2 strata

In 871 patients presumed F0F1F2 with FS, FT was associated with LSM (R=0.38; P<0.00001), with AT (R=0.51; P<0.00001) and with ST (R=0.15; P=0.0002). Discordant patients (possible FS false negative or FT false positive) (n=178) vs concordant (n=692) had higher AT (0.47 95%CI 0.43-0.50 vs 0.24 95%CI 0.23-0.26; P<0.00001) and no difference between ST. When adjusted on LSM (partial correlation) FT was still associated with AT (R=0.44; P<0.00001), and borderline with ST (R=0.08; P=0.048).

F0F1F2F3 strata

In 991 patients presumed F0F1F2F3 with FS, FT was associated with LSM (R=0.45; P<0.00001), with AT (R=0.54; P<0.00001) and with ST (R=0.15; P=0.00007). Discordant patients (possible FS false negative or FT false positive) (n=102) vs concordant (n=889) had higher AT (0.62 95%CI 0.57-0.66 vs 0.28 95%CI 0.26-0.29; P<0.00001) and higher ST (0.40 95%CI 0.35-0.45 vs 0.33 95%CI 0.31-0.34; P=0.01). When adjusted on LSM (partial correlation) FT was still associated with AT (R=0.45; P<0.00001), and not with ST (R=0.03; P=0.38).

F3F4 strata

In 238 patients presumed F3F4 with FS, FT was associated with LSM (R=0.48; P<0.00001), with AT (R=0.42; P<0.00001) and with ST (R=0.50; P<0.00001). Discordant patients (possible FS false negative or FT false positive) (n=63) vs concordant (n=175) had higher AT (0.59 95%CI 0.55-0.62 vs 0.34 95%CI 0.27-0.40; P<0.00001) and no difference between ST. When adjusted on LSM (partial correlation) FT was still associated with AT (R=0.44; P<0.00001), and not with ST (R=-0.16; P=0.06).
