# Supplementary material for: Concordance in a World without a Gold Standard: A New Non-Invasive Methodology for Improving Accuracy of Fibrosis Markers
Source: PLoS One. 2008 Dec 4;3(12):e3857. doi: 10.1371/journal.pone.0003857 (PMC2586659; doi:10.1371/journal.pone.0003857)
Supplement: Text S5 — Confounding variables (0.03 MB DOC) [file pone.0003857.s012.doc]

***Supporting Text S5: Confounding variables***

*Association between LSM, FT and potential variability factors*

As expected, LSM and FT were highly associated (all R>= 0.57; P<0.00001) with known risk factors of fibrosis (which could also be variability factors of LSM): age, male gender, waist circumference, serum glucose, necroinflammatory grades presumed with ActiTest, and steatosis presumed with SteatoTest. LSM, but not FT, was associated with weight (P<0.000001) and BMI (P=0.000002).

*Intra-association between manufacturers quality criteria*

We observed a high association between IQR/LSM and LSM, R=0.70 (n=2004). Among patients with advanced fibrosis (defined as LSM greater than 8.8 kPa) 30% (168/561) had a non-recommended dispersion of LSM (IQR/LSM>30%), which was twice that in patients without advanced fibrosis (defined as LSM lower than 8.8 kPa): 15% (213/1443) (P<0.0001).

Among patients with the recommended criteria and concordant operators (n=1,109) we still observed a significant and positive association between LSM and IQR/LSM (P<0.000001).

*Intra-association between potential variability factors*

When a factor was associated with variability without a clear rationale, one relevant hypothesis was an association with another variable (confounding factor).

Older age was significantly associated with lower concordance and this could be related to the following more rational factors that are also significantly associated with age: NAFLD, thoracic fold, waist circumference, BMI, serum glucose, ST, and AT. Among 343 patients without presumed fibrosis using FT, the correlation between LSM and age, decreased similarly from R=0.19 (P=0.0005) to pR=0.11 (P=0.02) after adjustment on BMI or ST. Age was positively associated with thoracic fold, waist circumference, serum glucose, ST (all P<0.000001), BMI (P=0.0002), AT (P=0.00008), female gender (P=0.001), negatively associated with height (P<0.000001) and thoracic fold (P=0.01).

Male gender was also positively associated with NAFLD, waist circumference, weight, BMI, AT, ST, alcohol consumption, and serum glucose. In patients without fibrosis using FT, the LSM was higher in male [n=267; 6.0 kPa (0.1)] than in female [n=233; 5.2 kPa (0.1)], but there were also a significant gender differences for confounding factors: BMI [24.9 (0.2) vs 23.5 kg/m2 (0.3); P=0.0005] and for ST [0.38 (0.02) vs 0.26 (0.02); P<0.00001] for male and female respectively.

Male gender was positively associated with waist circumference, height, weight, BMI, AT, ST, (all P<0.000001), alcohol consumption (P=0.00005), serum glucose (P=0.0004), and negatively associated with age (P=0.001).

NAFLD, in comparison with other causes of liver disease, was associated with weight, abdominal fold, thoracic fold, waist circumference, serum glucose, age, ST (all P<0.000001), male gender (P=0.02), and negatively with success rate (P=0.00003),

*Association between LSM, FT and potential variability factors*

As expected LSM and FT were highly associated (all R>= 0.57; P<0.00001) with known risk factors of fibrosis (which could also be variability factors of LSM): age, male gender, waist circumference, serum glucose, necroinflammatory grades presumed with ActiTest, and steatosis presumed with SteatoTest. LSM and FT were also associated with alcohol consumption (P=0.001; P=0.03).

LSM but not FT was associated with height (P=0.004), weight (P<0.000001) and BMI (P=0.000002).

*Inter-association between quality criteria and potential variability factors*

IQR/LSM was positively associated (P<0.004) with all the known fibrosis risk factors: age, male gender, waist circumference, serum glucose, AT, and ST. In multivariate analysis only male gender was associated with IQR/LSM (P=0.01).

Success rate was strongly and negatively associated with abdominal fold, thoracic fold, waist circumference, weight, and BMI (all P<0.000001); there was also a negative association between success rate and age (P<0.000001), ST (P=0.0002), and serum glucose (P=0.001). Success rate was not associated with gender (P=0.50), with FT (P=0.99) or AT (P=0.05).

In multivariate analysis, only thoracic fold (P=0.00009), age (P=0.002) and weight (P=0.01) were significantly associated with success rate.
